# APOE2 protects against Aβ pathology by improving neuronal mitochondrial function through ERRα signaling

**DOI:** 10.1186/s11658-024-00600-x

**Published:** 2024-06-12

**Authors:** Zhiyuan Ning, Ying Liu, Mengyao Wan, You Zuo, Siqi Chen, Zhongshan Shi, Yongteng Xu, Honghong Li, Ho Ko, Jing Zhang, Songhua Xiao, Daji Guo, Yamei Tang

**Affiliations:** 1 Department of Neurology, Sun Yat-Sen Memorial Hospital, Sun Yat-Sen University, Guangzhou, 510120 China; 2 Brain Research Center, Sun Yat-Sen Memorial Hospital, Sun Yat-Sen University, Guangzhou, 510120 China; 3 Nanhai Translational Innovation Center of Precision Immunology, Sun Yat-Sen Memorial Hospital, Foshan, 528200 China; 4 Division of Neurology, Department of Medicine and Therapeutics & Li Ka Shing Institute of Health Sciences, Faculty of Medicine, The Chinese University of Hong Kong, Shatin, Hong Kong, China; 5 Department of Neurology, Fujian Medical University Union Hospital, Fujian Key Laboratory of Molecular Neurology and Institute of Neuroscience, Fujian Medical University, Fuzhou, China; 6 Guangdong Provincial Key Laboratory of Malignant Tumor Epigenetics and Gene Regulation, Guangdong-Hong Kong Joint Laboratory for RNA Medicine, Medical Research Center, Sun Yat-Sen Memorial Hospital, Sun Yat-Sen University, Guangzhou, 510120 China; 7 Guangdong Province Key Laboratory of Brain Function and Disease, Zhongshan School of Medicine, Sun Yat-Sen University, Guangzhou, 510080 China

**Keywords:** Alzheimer's disease, Apolipoprotein E, Beta-amyloid (Aβ), Neuron, Mitochondria, ESRRA

## Abstract

**Background:**

Alzheimer’s disease (AD) is a progressive neurodegenerative disease and apolipoprotein E (APOE) genotypes (APOE2, APOE3, and APOE4) show different AD susceptibility. Previous studies indicated that individuals carrying the APOE2 allele reduce the risk of developing AD, which may be attributed to the potential neuroprotective role of APOE2. However, the mechanisms underlying the protective effects of APOE2 is still unclear.

**Methods:**

We analyzed single-nucleus RNA sequencing and bulk RNA sequencing data of APOE2 and APOE3 carriers from the Religious Orders Study and Memory and Aging Project (ROSMAP) cohort. We validated the findings in SH-SY5Y cells and AD model mice by evaluating mitochondrial functions and cognitive behaviors respectively.

**Results:**

The pathway analysis of six major cell types revealed a strong association between APOE2 and cellular stress and energy metabolism, particularly in excitatory and inhibitory neurons, which was found to be more pronounced in the presence of beta-amyloid (Aβ). Moreover, APOE2 overexpression alleviates Aβ1-42-induced mitochondrial dysfunction and reduces the generation of reactive oxygen species in SH-SY5Y cells. These protective effects may be due to ApoE2 interacting with estrogen-related receptor alpha (ERRα). ERRα overexpression by plasmids or activation by agonist was also found to show similar mitochondrial protective effects in Aβ1-42-stimulated SH-SY5Y cells. Additionally, ERRα agonist treatment improve the cognitive performance of Aβ injected mice in both Y maze and novel object recognition tests. ERRα agonist treatment increased PSD95 expression in the cortex of agonist-treated-AD mice.

**Conclusions:**

APOE2 appears to enhance neural mitochondrial function via the activation of ERRα signaling, which may be the protective effect of APOE2 to treat AD.

**Supplementary Information:**

The online version contains supplementary material available at 10.1186/s11658-024-00600-x.

## Introduction

Alzheimer’s disease (AD), a type of progressive neurodegenerative disorder, is the most common cause of dementia and is an urgent global issue to be addressed. The apolipoprotein E (APOE) genotype, a notable genetic factor for late-onset AD, varies at amino acids at positions 112 and 158, resulting in three prevalent alleles (APOE2, APOE3, and APOE4) [1]. Of the three APOE alleles, APOE2 tends to lower the risk of AD, while APOE4 increases the risk relative to APOE3 [2]. The different effects of APOE2 and APOE4 on AD risk may be attributed to some similar mechanisms, such as beta-amyloid (Aβ) aggregation and clearance and neurofibrillary tangle formation [3, 4]. Studies on non-demented elderly individuals and AD patients have demonstrated the association between APOE2 and reduced Aβ deposition in the brains [3–5], suggesting that APOE2 partially mitigates AD risk through an Aβ-dependent manner. However, few studies examined the protective role of APOE2 in AD-related pathology [6], but several findings showed that APOE2 may confer protection by regulating lipid metabolism and synaptic functions [7–9]. Although the APOE4's mechanism has been extensively studied for AD risk, comprehension of the biological basis for protective effects of APOE2 remains limited. Understanding the unique properties and potential benefits of APOE2 is crucial for developing novel strategies for the prevention and treatment of AD.

The precise pathogenetic mechanism of AD remains elusive. Substantial evidence suggests that mitochondrial dysfunction may contribute to the pathophysiology of AD. Mitochondrial dysfunction and aberrations in brain energy metabolism may manifest even in the preclinical stage of AD [10]. Moreover, amyloid plaques may cause mitochondrial damage, and dysfunctional mitochondria may lead to abnormal amyloid protein metabolism [10]. Healthy mitochondria not only provide energy for neuronal activity, but they also protect neurons against from oxidative damage [11]. Furthermore, treatments that target mitochondrial oxidative stress have been proven to alleviate AD-related pathologies [12]. Consequently, mitochondria-centric treatments are being considered as potentially effective AD therapies. Numerous ongoing clinical trials are exploring treatments focusing on mitochondrial or related pathways, such as antioxidants, mitochondrial enhancers, and anti-apoptotic drugs [12]. These findings suggest that interventions aimed at mitochondrial functions present a promising therapeutic approach, particularly for early intervention of disease.

Individuals without cognitive impairments but with a risk for AD, particularly those carrying the APOE4, exhibit reduced brain glucose metabolism [13, 14]. Additionally, research indicates that mice with the APOE2 gene display enhanced activity in glucose transport and insulin-like growth factor 1 signaling [15]. Notably, individuals with APOE2 have higher brain metabolism compared to those with APOE3 or APOE4. Increasing evidence suggests the APOE genotype affects mitochondrial and endoplasmic reticulum stress differently depending on the APOE variant. Previous study has shown that the mitochondria-associated PPAR-γ/PGC-1α signaling pathway is activated in APOE2-expressing brains while inhibited in APOE4 brains. Enhancing PGC-1α expression seems to counteract issues in glycolysis and mitochondrial function caused by APOE4 [16]. These findings highlight the influence of APOE genetics on brain energy metabolism, though the exact mechanisms remain to be fully understood.

In this study, we performed differential pathway activity analysis using single-nucleus RNA sequencing and bulk RNA sequencing data of brain tissue from individuals pathologically confirmed AD and age-matched control. Based on the APOE genotypes, our analysis identified genes and biological pathways related to mitochondrial function of neurons, which might be involved in the protective effect of APOE2. Overexpressing APOE2 in SH-SY5Y could alleviate Aβ1-42-induced mitochondrial dysfunction and reduce the generation of reactive oxygen species. Moreover, ApoE2 showed protective effect by interacting with estrogen-related receptor alpha (ERRα). ERRα overexpression or activation could also restore the mitochondrial function in Aβ1-42-stimulated SH-SY5Y cells. Significantly, ERRα activation agonist treatment improves the cognitive performance in mice with intracerebroventricular injection of Aβ1-42. Together, our findings reveal a previously undescribed ApoE-ERRα-dependent mechanism underlying the neural mitochondrial function, which is important on AD pathology.

## Methods

### Datasets

Data of single-nucleus RNA sequencing (snRNA-Seq) and bulk RNA-sequencing (bulk RNA-Seq) were obtained from the ROSMAP cohort. In the snRNA-Seq dataset from dorsolateral prefrontal cortex (DLPFC) in Experiment 2 (syn31512863: https://www.synapse.org/#!Synapse:syn31512863), a random selection of 52 samples possessing either APOE2/3 or APOE3/3 genetic types were extracted from a pool of 465 unique donors [17]. In addition, for the snRNA-Seq data obtained from Mathys et al. (syn21261143: https://www.synapse.org/#!Synapse: syn21261143), a total of 34 samples with APOE2/3 or APOE3/3 genotypes were included in the analysis [18]. For the bulk RNA-Seq data (syn3388564: https://www.synapse.org/#!Synapse:syn3388564), an assemblage of 470 samples linked to APOE2 or APOE3 genetic types were chosen for this study [19]. The detail information of all samples is listed in the Supplementary Table S1.

### Single-nucleus RNA-seq data processing

Quality control procedures involved the following preset filters in Seurat [20]: (1) Removing cells with less than 500 or more than 10,000 identified genes; (2) Excluding genes that were expressed in fewer than ten cells. We focused only on protein-coding genes for further analysis; and (3) Using a k-means clustering approach to binarize data, identifying and excluding cells that exhibit a high ratio of mitochondrial to non-mitochondrial read counts. After QC, we retained 102,316 nuclei and 17,829 genes for subsequent analysis. We then performed log-normalization, scaling, and dimension reduction and visualization within the Seurat workflow. To correct for batch effects, we applied Harmony [21]. We used ACTIONet to annotate cell types by referencing previously documented gene expression patterns from the prefrontal cortex (PFC) regions [22]. SAVER, an expression recovery method, was utilized to recover the drop-out genes in neuronal cells [23].

### GSVA pathway analyses

#### Databases

The GO_Biological_Process_2021 pathway database consisting of 6036 pathways was downloaded from the Maayan laboratory (https://maayanlab.cloud/Enrichr/#libraries). A total of 138 mitochondrial-associated pathways were curated by filtering the union of pathways from GO_Biological_Process_2021, KEGG_2021_Human, Reactome_2022 and HumanCyc_2016 databases. Gene sets featuring at least one of the following terms in their name: oxygen, mitochondrial, energy, respiration, glycolytic, NADH, oxidative phosphorylation, ATP, aerobic respiration, electron transfer and electron transport, were selected. All mitochondrial-associated pathways and their genes can be found in Supplementary Table S3. The 'MITOMAP: Nuclear Mitochondrial Genes' resource supplemented the pathway information, and was accessed via http://www.gen.emory.edu/mitomap.html. The MitoCarta3.0 database was accessed via https://www.broadinstitute.org/mitocarta/mitocarta30-inventory-mammalian-mitochondrial-proteins-and-pathways.

#### Pathway activity score calculation

The average normalized gene expression profiles for each cell type were first computed at the individual level and then calculated the pathway activity scores using the R package GSVA (v.1.42.0) [24]. GSVA estimates normalized gene expression levels across samples, ranks these levels, and aggregates them into gene sets based on enrichment scores derived from a Kolmogorov–Smirnov-like metric. The GSVA function was executed with parameters: mx.diff = TRUE, kcdf = "Gaussian", min.sz = 5, and max.sz = 150.

#### Differential pathway activity analysis

To reduce false detections, we only considered genes with at least a 10% detection rate of non-zero values in certain cells. We analyzed pathway activities in each cell type by employing a multivariate linear model: pathway activity ~ β0 × APOE2 + β1 × age_death + β2 × pmi + β3 × sex + β4 × pathological_diagnosis_of_AD. The linear models were created using the Limma R package. Pathways influenced by APOE2 and showing a *P* value below 0.05 were selected for further investigation.

### irGSEA pathways analyses

We utilized the irGSEA (v1.3.3) R package to evaluate the pathway activity scores of each cell. The package was used to calculate enrichment scores among each cell type for inputted pathways via the AUcell function and identify differentially expressed gene sets using the Wilcoxon test.

### Differential gene expression

The 'FindMarkers' function in the Seurat package was deployed to identify genes with differential expression, applying criteria of an absolute log_2_ fold change (FC) greater than 0.1 and an adjusted p-value below 0.05. These identified genes underwent GO enrichment analysis using the clusterProfiler package. Genes overlapping with the mitocarta 3.0 database were classified as mitochondria-related DEGs.

### Identification of APOE2-associated cells

Scissor software (version 2.1.0) was employed to discern APOE2-associated cell subpopulations within the snRNA-Seq data by utilizing the phenotypes collected from bulk assays [25]. Scissor learned the distinct expression features found in bulk RNA-Seq data through logistic regression for binomial data and identify the key cells in snRNA-Seq data using Pearson correlation. Our study applied bulk RNA-Seq data from ROSMAP and set a filter threshold of 0.2 to identify robust cells linked to the APOE2 genotype, leading to the prediction that cells marked as Scissor^+^ are likely associated with the APOE2 genotype.

### Gene regulatory network construction

To optimize computational efficiency, we initially sampled 10,000 neuronal cells using the Seurat sample function prior to analysis. The pySCENIC tool was employed to assess the enrichment of transcriptome factors (TF) in each cell. In brief, the standard SCENIC procedure encompasses three steps: (1) identification of candidate regulatory modules based on coexpression patterns between genes; (2) refinement of coexpression modules by eliminating indirect targets using transcription factors motif information; and (3) measurement of the activity of these discovered regulons in individual cells [26]. To compare the differences in neuronal transcription factor activity between the APOE2 and the APOE3 populations, we employed the limma R package for differential analysis. Transcription factors with an adjusted *p*-value < 0.05 were considered as potential candidates. Lastly SCENIC analysis was utilized to predict the downstream target genes of ESRRA.

### Molecular docking

The ApoE3 crystal structure was acquired from the RCSB Protein Data Bank (PDB code: 2L7B, NMR structure of full-length ApoE3). The structure of ApoE2 was modeled by mutating the ApoE3 structure using PyMOL [27]. ERRα was predicted by AlphaFold2 using the full length of amino acid sequences obtained from the RCSB Protein Database Bank [28]. The binding interaction between ApoE2 or ApoE3 and ERRα was investigated through a docking study using the HDOCK server [29]. A lower docking score from this server indicates a more precise prediction of the binding mode. Visualization, analysis, and mapping of the best predicted binding mode were carried out using the Discovery Studio 2021 and Visual Molecular Dynamics (VMD) program. This facilitated the display of key residues of ApoE2 or ApoE3 and ERRα.

### Animals

Adult ICR mice, comprising an equal number of males and females aged between 8 and 12 weeks, were obtained from the Laboratory Animal Center at Sun Yat-Sen University. hAPOE2 and hAPOE3-TR mice were acquired from the laboratory of Professor Jing Zhang. All mice were kept in a Specific Pathogen Free (SPF) environment. The study received ethical approval from the Ethics Committee of Sun Yat-Sen University, under the Approval number 2022000485.

### Cell culture

SH-SY5Y cells (Servicebio, China, STCC11004P) and HEK-293T cells (Servicebio, China, STCC10301P) were cultured in DMEM/F12 medium (Gibco, Carlsbad, CA, USA) and DMEM medium (Gibco, Carlsbad, CA, USA) respectively, enriched with 10% heat-inactivated fetal bovine serum (Procell, Wuhan, China) and 1% penicillin/streptomycin (Biosharp, Hefei, China). All experiments were conducted 24 h after seeding. Cells used for fluorescent cell imaging were grown in 48-well cell culture plates, those used for cell viability assays were grown in 96-well plates and those used for western blot were grown in 12-well plates (All from Corning, USA). Cells for microplate reader testing were grown in all-white 96-well cell culture plates (FCP968, Beyotime, China).

### Plasmid preparation and transfection

Human APOE2 (CMV-human APOE2-3FLAG-SV40-Puromycin, CMV-human APOE2-HA-SV40-Puromycin) and APOE3 (CMV-human APOE3-MYC-SV40-Puromycin) overexpression plasmids were obtained from Genechem (Shanghai, China). Human ESRRA overexpression plasmids (CMV-ESRRA (human)-3FLAG-SV40-Neo, based on full-length human ESRRA as a template [30]) were got from MiaoLing (Wuhan, China). Lipofectamine™ 3000 (Thermo Fisher Scientific, Waltham, MA, USA) was used to transfect SH-SY5Y cells with the plasmids when the fusion rate reached 60–70%. Following transfection, the culture medium was changed every 8 h, and the detection of related proteins or subsequent treatments were performed 48 h after transfection. Plasmid overexpression was verified through immunoblotting (Fig. S6 c, d).

### Pharmacological treatment of SH-SY5Y cells

Β-amyloid (Aβ) oligomers were prepared from human Aβ (1–42) peptide (RP10017CN, GenScript, New Jersey, USA) following a previously established protocol [31, 32]. The β-Amyloid (1–42) peptide was initially dissolved in hexafluoroisopropanol (HFIP, H811027-2 ml, Macklin, Shanghai, China) at a concentration of 1 mM and incubated for over 30 min at room temperature. The solution was then divided into sterile microcentrifuge tubes as aliquots. HFIP was subsequently removed using a vacuum freeze dryer (Labconco™ FreeZone™ 2.5L). To prepare Aβ oligomers, the peptide was dissolved in DMSO (D2650, Sigma-Aldrich, USA) to a concentration of 5 mM and then diluted in PBS to a final concentration of 100 μM. The mixture was incubated for over 24 h at 4 °C. SH-SY5Y cells were treated with Aβ oligomers (10 μM) 48 h after plasmid transfection.

1-[4-(3-tert-Butyl-4-hydroxyphenox) phenyl] ethan-1-one, an ERRα agonist (HY-132205, MedChemExpress, USA) was selected based on the study conducted by Shinozuka T [33]. SH-SY5Y cells were treated with the ERRα agonist at a concentration of 5 μM.

### Measurement of mitochondrial functions

#### Measurement of mitochondrial membrane potential (MMP)

Measurement of mitochondrial membrane potential (MMP) was detected using fluorescent probe JC-1 (BL711A, Biosharp, Hefei, China) and the MitoTracker Red CMXRos (40741ES50, Yeasen, Shanghai, China).

JC-1 is a dye that reacts to changes in mitochondrial transmembrane potential (ΔΨm). In healthy cells, it forms J-aggregates emitting red/orange fluorescence when ΔΨm is high, and green fluorescence as ΔΨm decreases. After treatment, cells were incubated with JC-1 at 37 °C for 20 min, washed twice with a buffer, and their fluorescence was observed under a microscope. We calculated ΔΨm as the ratio of red JC-1 aggregates to green JC-1 monomers. Carbonyl cyanide 3-chlorophenylhydrazone (CCCP) was served as the negative control. ImageJ software was used for fluorescence intensity calculations.

MitoTracker Red CMXRos labels functional mitochondria for relative MMP detection. SH-SY5Y cells were incubated with 1 μM MitoTracker Red at 37 °C for 30 min, and washed with PBS. Fluorescence levels were quantified with a modular multimode microplate reader (Biotek SynergyH4, USA). Cells were then fixed in 4% paraformaldehyde and observed under a fluorescent microscope. ImageJ software was used for fluorescence intensity calculations.

#### Measurement of mitochondrial ROS level

Mitochondrial ROS were detected using the MitoSOX Red probe (HY-D1055, MedChemExpress, New Jersey, USA). SH-SY5Y cells were incubation with 5 μM MitoSOX Red working solution at 37 °C for 30 min in the dark, following by wash using PBS. The ensuing step involved the quantification of relative fluorescence levels either via a modular multimode microplate reader (Biotek SynergyH4, US) or a fluorescence microscopy coupled with the use of ImageJ software for measurement.

### Immunofluorescence staining of brain tissue

Immunofluorescence staining was used to verify the localization of the ERRα, the neuronal marker NeuN, and ApoE in adult mouse brain. The brains were dissected, fixed in 4% paraformaldehyde, and sliced into 20 μm thick sections using a vibratome (Leica CM1950). These sections were then blocked for 2 h at room temperature using a solution containing 0.3% Triton X-100, 5% normal donkey serum, and 5% BSA. After blocking, the sections were incubated with primary antibodies overnight at 4 °C and then incubated with secondary antibodies for 2 h at room temperature. Then, the immunofluorescence staining was observed using an Olympus BX63 microscope. The primary antibodies used were mouse-anti-NeuN antibody (ab104224, Abcam, the UK, 1:1000), rabbit-anti-ERRα antibody (ab76228, Abcam, the UK, 1:100), and goat-anti-Apolipoprotein E antibody (178,479-500ULCN, MERCK, Germany, 1:500).

### Co-immunoprecipitation assay

Co-IP in HEK-293 T cells was performed using Anti-HA-Tag Mouse Antibody (Agarose Conjugated) (Abmart, Shanghai, China, M20013) according to the instructions. Cells were lysed with 1 × LysisWash Buffer and then centrifuged to separate the cellular debris. A total of 500 µg of protein was incubated with 25 µl of the antibody with gentle mixing for 14 h at 4 °C. The precipitated proteins were eluted from the beads by adding elution buffer. Finally, the eluted proteins were subjected to western blotting analysis. The antibodies used for Co-IP incubation with proteins were mouse-anti-FLAG antibody (MBL, Nagoya, Japan, M185-3) and mouse-anti-HA antibody (Proteintech, Wuhan, China, 66,006–2-ig). Co-IP in the mouse brain cortex was performed using the rProtein A/G Magnetic IP/Co-IP Kit (ACE, Changzhou, China, BK0004-01). According to the instructions, 1.5 mg of brain tissue was used, and the experiment was conducted with 10ug of APOE antibody or IgG.

### Western blotting

Cells and brain samples were lysed on ice with RIPA lysis buffer (P0013B, Beyotime, China). The supernatants were collected, and their protein levels were measured using the BCA assay. Proteins (about 20 μg) were then separated by SDS–polyacrylamide gel electrophoresis and transferred to PVDF membranes. The membranes were then incubated overnight at 4 °C with ERRα antibody (ab76228, Abcam, the UK, 1:1000), PSD95 (ab12093, Abcam, the UK, 1:2000), β-tubulin (66,240–1-ig, proteintech, China, 1:5000), and α-Actinin (66,895–1-Ig, proteintech, China, 1:5000), Synaptophysin (ab32127, Abcam, the UK, 1:2000) and Homer1 (ab184955, Abcam, the UK, 1:2000), and subsequently with HRP-conjugated secondary antibodies. Detection was performed using the Odyssey Infrared Imaging System (LI-COR, Biosciences, Lincoln, NE, USA).

### CCK8 assay for cell viability after ERRΑ agonist treatment

Cell viability was measured using an enhanced Cell counting kit 8 (CCK-8) assay (GOONIE). SH-SY5Y cells were plated in 96-well plates at a density of 5000 cells per well and then treated with varying concentrations of ERRα agonist for 48 h. At the specified time point, CCK-8 mixture was incubated for 1 h and then cells were detected by a modular multimode microplate reader (BioTek Synergy H1, USA).

### Induction of the AD-model

Mice were IP anaesthetized with 0.2 ml/10 g avertin (M2910, Aibei Biotechnology, Nanjing, China). Aβ1-42 peptide (80 pmol/ul, dissolved in sterile PBS) was injected to the intracerebroventricular to induce AD-model with a delivery rate of 1 μl/min, totaling 5 μl [34].

### Nasal administration of ERRα agonist

ERRα agonist were administered to the mice nasally at 3 and 6 days following the induction of the AD-model. To improve absorption, we first applied 50U of hyaluronidase (sourced from Macklin, China, product code H6151) into each nostril half an hour before nasal delivery of agonist. The dose of ERRα agonist was 1 mg/kg body weight, given in alternating nostrils, 3 µl at a time, amounting to 12 µl in total for each mouse [35].

### Behavioral tests

#### Novel object recognition test

The novel object recognition test was conducted following established methods [36]. Initially, the mice were familiarized with an empty square chamber for 10 min on the first day. On the second day, they were introduced to two identical objects in the same chamber for a 10-min exploration period. On the third day, one of these objects was swapped with a new one, and the mice were allowed another 10 min to explore. The exploration time referred to how long a mouse spends sniffing an object from a distance of up to 2 cm, with its nose oriented straight towards the object. The recognition ratio was calculated by dividing the time spent sniffing one specific object by the total time spent sniffing both objects.

#### Y maze spontaneous alternation test

The Y Maze Spontaneous Alternation Test evaluates working memory through a unique maze setup. This Y-shaped maze has three arms, positioned at 120 degrees apart. Mice are placed at the maze's center and can explore for 10 min. An entry into an arm is counted when a mouse's tail tip fully enters. An alternation is noted when the mouse enters a different arm than its last two choices. The alternation rate is calculated as the number of alternations divided by the total arm entries minus two, expressed as a percentage.

### Statistical analysis

Data analysis was conducted using IBM SPSS Statistics. The presented data represents the results of at least three independent experiments, each performed in triplicate (*n* ≥ 3). Densitometry analysis for Western blot was performed using blots from at least three independent experiments (*n* ≥ 3). For parametric data, normal distribution and variances within each comparison group of data were checked before analyses. Statistical analysis was carried out using one-way ANOVA followed by Tukey post-hoc test or Holm-Šídák's multiple comparisons test. Kruskal–Wallis test was used in the data with non-normal data distribution. The error bars indicated the standard error of the mean (SEM). Statistical significance levels were denoted as follows: *P < 0.05; **P < 0.01; ***P < 0.001; ****P < 0.0001; ns, not statistically significant (*P* > 0.05). Correlation analysis was conducted using the stat_cor() function in the ggpubr R package, and statistical differences between correlations were assessed using the Cocor [37].

## Results

### APOE2 single-nucleus profiling and cellular diversity

**Fig. 1:**
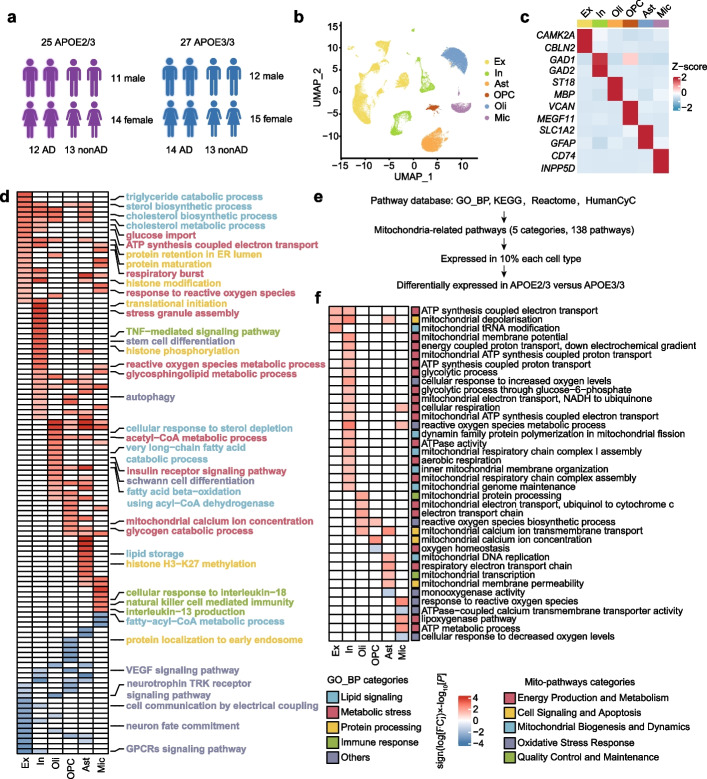
APOE2 single-nucleus profiling and pathway-level alterations. **a** Detailed information of APOE carriers from The Religious Orders Study and Memory and Aging Project (ROSMAP) (Created with https://www.biorender.com). **b** The UMAP plot displayed six main cell types in DLPFC. Ex: excitatory neurons; In: inhibitory neurons; Oli: oligodendrocytes; OPC: oligodendrocyte precursor cells; Ast: astrocytes; Mic: microglia. **c** Heatmap showed the expression of representative marker genes in each cell type. **d** Heatmap showed top Gene Ontology biological processes with expression changes associated with APOE2 (nominal *P* < 0.05, linear model, APOE32/3 versus APOE3/3), with red indicating APOE2 upregulation and blue indicating APOE2 downregulation. The color scale represented sign (log [FC]) × log10[*P*] values. **e** The curation process of mitochondrion-associated pathways, GO_BP, GO_Biological_Process_2021 database; KEGG, KEGG_2021_Human database; Reactome: Reactome_2022 database; HumanCyc: HumanCyc_2016 database. **f** Heatmap showed mitochondrion-associated pathways altered across major six cell types in APOE2 versus APOE3 individual (nominal *P* < 0.05, linear model). Red indicated APOE2 upregulation and blue indicated APOE2 downregulation. The color scale represents sign (log [FC]) × log10[P] values. Pathways with absolute value of sign (log [FC]) × log10[*P*] > 1.3 were shown in the heatmap

To examine the effect of APOE2 in the aging human brain, we investigated the single-nucleus RNA sequencing dataset of 52 individuals, which included 25 APOE2/3-carriers and 27 APOE3/3-carriers, randomly chosen from the ROSMAP. Among the 52 individuals, half exhibited high levels of β-amyloid and other pathological hallmarks of Alzheimer’s disease ('AD-pathology', 'AD'), while the remaining half showed either no or low β-amyloid burden ('non-AD-pathology', 'nonAD'), which aligned with the NIA-AA criteria [18, 38, 39]. Both APOE3/3 and APOE2/3 groups maintained a balance in terms of AD pathology, gender (APOE2/3: 11 males and 14 females, APOE3/3: 12 males and 15 females), age (median: 86.78 APOE2/3, 85.76 APOE3/3), years of education (median: 16.20 APOE2/3, 16.04 APOE3/3), and post mortem interval (PMI) (median: 7.245 APOE2/3, 8.026 APOE3/3) (Fig. 1a). Detailed clinical data are provided in Supplementary Table S1.

After pre-processing, the single-nucleus RNA sequencing dataset was found to contain 17,829 genes across 102,316 nuclei (Fig. 1b). As demonstrated by the UMAP graph and the heatmap, these nuclei were assigned to 6 main cell types according to established gene markers [40, 41] including excitatory neurons (Ex, CAMK2A, CBLN2), inhibitory neurons (In, GAD1, GAD2), astrocytes (Ast, SLC1A2, GFAP), microglia (Mic, CD74, INPP5D), oligodendrocytes (Oli, ST18, MBP) and oligodendrocyte precursor cells (OPC, VCAN, MEGF11) (Fig. 1b, c). Vascular cells and peripheral immune cells were excluded considering that they have a small number and are not present in each sample.

### APOE2 pathway-level alterations

We investigated the transcriptomic effects of APOE2 on molecular processes by calculating pathway activity scores for each individual and cell type. This was done by combining pseudo-bulk approaches with Gene Set Variation Analysis (GSVA), focusing on Gene Ontology biological processes. The scores for APOE2/3 and APOE3/3 pathways were compared using a multivariate linear model, which adjusted for factors such as sex, post-mortem interval (PMI), age at death, and CERAD score. This analysis identified 111 potential APOE2-associated molecular processes (shown in Fig. 1d), each with a nominal *P* value of less than 0.05, as determined by the linear model comparing APOE2/3 to APOE3/3.

In APOE2 carriers, we observed several cell-type-specific alterations in biological processes (Fig. 1d; Supplementary Table S2, a detailed atlas of APOE2-related pathway changes in each cell type). Immune-related pathways (marked in green), including response to interleukin-18 and natural killer cell mediated immunity, were upregulated in microglia. In inhibitory neurons, there was an increase in tumor necrosis factor (TNF)-mediated signaling (Fig. 1d). Additionally, protein processing pathways (marked in yellow) varied across cell types; for instance, upregulation of protein retention in the ER lumen and protein maturation in neurons, and enhanced histone modification and histone H3-K27 methylation in astrocytes were noted. We also detected a decrease in processes linked to growth and nutritional factors, including the vascular endothelial growth factor signaling pathway and neurotrophin TRK receptor signaling pathway (Fig. 1d). Notably, these alterations in immune response, protein processing, and other pathways were evident not only in glial cells, typically most affected by APOE genotypes, but also in neurons.

Importantly, APOE2 was associated with energy metabolism in multiple cell types. A number of pathways related to lipid signaling (marked in blue) and metabolic stress (marked in red) were changed almost in all six cell types. For example, triglyceride catabolic process was enhanced in excitatory neurons, cholesterol biosynthetic and metabolic process were upregulated among neurons, oligodendrocytes, and astrocytes, while fatty-acyl-CoA metabolic process was downregulated in microglia (Fig. 1d). Remarkably, we observed a substantial increase in the number of mitochondrial energy metabolism pathways in APOE2-carriers. Glucose imports and ATP synthesis coupled electron transport were enhanced in both types of neurons, regulation of respiratory burst and reactive oxygen species were upregulated in neurons and microglia, acetyl-CoA metabolic and glycogen catabolic process were strengthened in both astrocytes and OPC (Fig. 1d). Consequently, the regulation of cellular metabolism, including mitochondrial bioenergetics and energy production, may serve as central protective mechanisms of APOE2.

### APOE2 influences neuronal mitochondrial function especially in the presence of AD pathology

We further analyzed the different pathways associated with mitochondrion across six major cell types in APOE2 and APOE3 individuals. A selection of 5 categories, 138 mitochondrial-associated pathways, from gene sets containing at least one of the following terms: oxygen, mitochondrial, energy, respiration, glycolytic, NADH, oxidative phosphorylation, ATP, aerobic respiration, electron transfer, and electron transport, were filtered from the union of pathways from different four pathway databases (Fig. 1e, Supplementary Table S3). Out of these, 40 mitochondrial-associated pathways showed alterations across different cell types, with 21 regulating neural function (Fig. 1f). Among these altered mitochondrial-related pathways, most of them are related to energy production and metabolism (marked in red), which suggests that APOE2 might be involved in neuronal mitochondrial energy metabolism (Fig. 1f, Supplementary Table S4).

To validate our findings, we further analyzed an additional single-nucleus RNA sequencing data containing the same brain region of 11 APOE2/3 and 23 APOE3/3 individuals [18]. As depicted by the UMAP plot, we also identified six primary cell types (Fig. S1a). Upregulated differentially expressed genes (DEGs) of excitatory and inhibitory neurons in APOE2/3 carriers showed a strong connection with mitochondrial functions in GO pathway analysis, when compared to who carries APOE3/3 (Fig. S1b, c). Moreover, we utilized Scissor to integrate the single-nucleus data and bulk transcriptome data of 470 individuals (86 APOE2 carriers and 384 APOE3 carriers) to identify APOE2-associated cells (Fig. S1d). The UMAP plot displayed APOE2 positive cells (cells positively correlated with APOE2 phenotypes), APOE2 negative cells (cells negatively correlated with APOE2 phenotypes), and background cells (cells uncorrelated with APOE2 phenotypes). The heatmap showed top20 marker genes of APOE2 positive cells, including inhibitory neuron markers (PVALB, GAD1, GAD2) and mitochondrial-related genes (Fig. S1e, f). Pathway analysis of these genes indicated their significant association with mitochondrial functions (Fig. S1g).

Overall, our results suggest that APOE2 is closely associated with cellular stress, as well as energy metabolism across various cell types, particularly in excitatory and inhibitory neurons, and that this association may be mediated through mitochondrial-related signaling pathways.

**Fig. 2:**
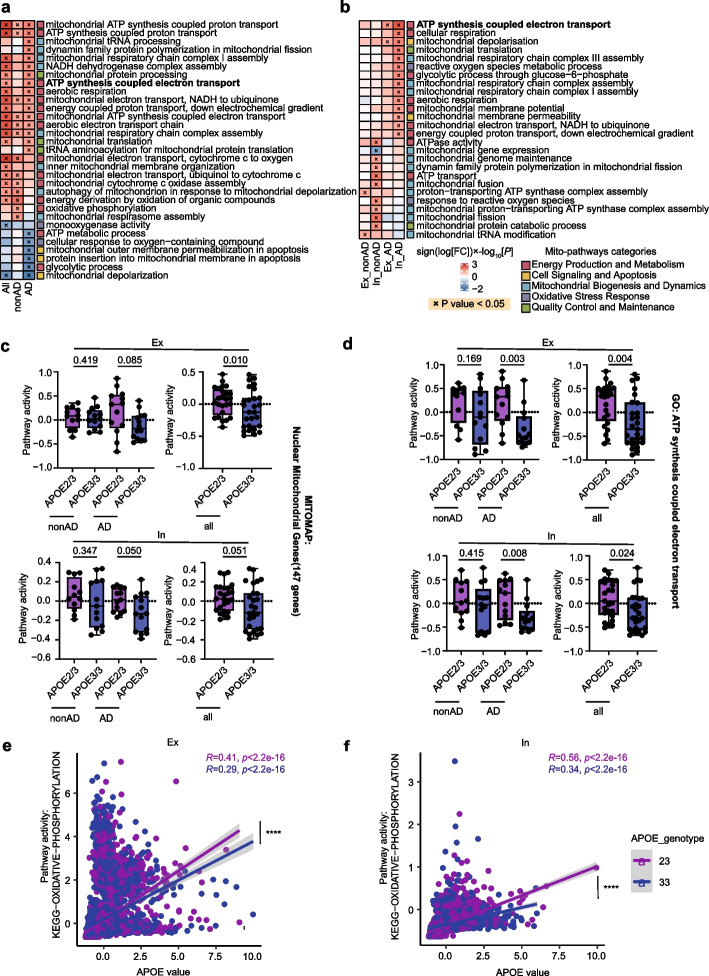
APOE2 drives mitochondrial changes in neurons especially when accompanied by AD pathology. **a**, **b** Heatmap showed mitochondrion-associated pathways altered in APOE2 versus APOE3 brain in bulk RNA-seq (**a**) and altered in excitatory and inhibitory neurons in APOE2 versus APOE3 individuals (**b**) stratified by amyloid. Red indicated APOE2 upregulation and blue indicated APOE2 downregulation. The color scale represents sign (log [FC]) × log10[*P*] values. *P* < 0.05 indicated (x). **c**, **d** Box plots showed pathway activity scores for ‘MITOMAP: Nuclear Mitochondrial Genes (147 genes associated with mitochondrion)’ (**c**) and for ‘GO: ATP synthesis coupled electron’(**d**) stratified by APOE genotype and/or AD pathology (nominal *P*-values, linear model). Boxplots indicates median, 25th and 75th percentiles. **e** Correlation analysis between oxidative phosphorylation pathway scores and the APOE expression values in excitatory neurons (Ex, APOE2: *R =* 0.41, *P* < 2.2e^−16^; APOE2: *R =* 0.29, *P* = 2.2e^−16^), with APOE2 excitatory neurons demonstrating a more significant correlation in comparison to APOE3 (*z* = 5.7837, *P* = 0.0000); ****P < 0.0001. **f** Correlation analysis between oxidative phosphorylation pathway scores and the APOE expression values in inhibitory neurons (In, APOE2: *R =* 0.56, *P* < 2.2e^−16^; APOE2: *R =* 0.34, *P* = 2.2e^−16^), with APOE2 inhibitory neurons demonstrating a more significant correlation in comparison to APOE3 (Z = 11.7636, *P* = 0.0000); ****P < 0.0001

A separate analysis of the bulk RNA-seq data echoed similar findings and further demonstrated that the dissimilarities in mitochondria-associated pathways between APOE2 and APOE3 brains were partially modified by the presence of Aβ [19] (Fig. 2a, Supplementary Table S5). Therefore, we delved into if Aβ pathology modified APOE2’s impact on neuronal mitochondria. Indeed, in excitatory and inhibitory neurons subject to Aβ, we noticed alterations in mitochondria-associated pathways. Among these, the ‘ATP synthesis coupled electron transport’ pathway appeared most significantly affected by APOE2 in both cell types of neurons in the presence of Aβ (Fig. 2b, Supplementary Table S6). Besides, we conducted differential mitochondria-related gene expression analysis on excitatory and inhibitory neurons of APOE2 and APOE3 carriers and found there was a higher number of DEGs in the presence of Aβ: 119 in excitatory neurons and 17 in inhibitory neurons, respectively (Fig. S2, Supplementary Table S7).

Additionally, we computed the pathway activity scores for ‘Nuclear Mitochondrial Genes (MITOMAP)’ (Fig. 2c) [42] and ‘GO: ATP synthesis coupled electron transport’ (Fig. 2d) in both excitatory and inhibitory neurons, considering the stratification by APOE genotypes and/or AD pathology. Our repeated findings suggest that APOE2 may, in comparison to APOE3 individuals, possess a more prominent ability to protect neuronal mitochondria from the damaging effects of amyloid pathology.

Given prior studies indicating a potential correlation between the neuronal APOE expression and the metabolic pathway module [43], we assigned oxidative phosphorylation pathway scores to each neuron utilizing AUCell and performed correlation analysis with the expression value of APOE. In line with previous findings, neuronal APOE expression displayed a positive correlation with the oxidative phosphorylation pathway score, irrespective of the APOE genotypes (Fig. 2e, f; excitatory neurons (Ex), APOE2/3: *R =* 0.41, *P* < 2.2e^−16^; APOE3/3: *R =* 0.29, *P* = 2.2e^−16^; inhibitory neurons (In), APOE2/3: *R =* 0.56, *P* < 2.2e^−16^; APOE3/3: *R =* 0.34, *P* = 2.2e^−16^). A comparative analysis of the correlation degree between APOE2 and APOE3 neurons was conducted utilizing the Cocor [37], revealing a stronger correlation in APOE2 neurons, including both excitatory and inhibitory neurons (Fig. 2e, f; excitatory neurons (Ex),: *Z* = 5.7837, *P* = 0.0000; inhibitory neurons (In): *Z* = 11.7636, *P* = 0.0000). These findings suggested that neuronal APOE2 could potentially exert greater influence on neuronal mitochondrial metabolism compared to APOE3.

Moreover, other factors like Braak stages, age and gender may relate to the enhanced neuronal mitochondrial pathway in APOE2 individuals. Therefore, we performed a correlation analysis between individual mitochondrial pathway scores in both excitatory and inhibitory neurons and their corresponding Braak stages. The resulting heatmap reveals a significant inverse correlation for many mitochondrial pathways with Braak stage progression in APOE3/3 individuals, such as ‘ATP synthesis coupled electron transport’ and ‘Oxidative phosphorylation’, which appear preserved in APOE2/3 individuals (Fig. S3a, b Supplementary Table S8). Additionally, our analysis of the relationship between mitochondrial pathway scores and age indicates that age might not be a primary factor in APOE2's mitochondrial protection (Fig. S3c, d, Supplementary Table S9). Regarding gender differences, we found that females exhibited a stronger protective effect of APOE2 in certain pathways, such as 'ATP synthesis coupled electron transport' and 'cellular respiration' (Figure. S3e, Supplementary Table S10).

We further focused on subtypes of neurons, where we identified seven cell types of excitatory neurons and five cell types of inhibitory neurons with their markers (Fig. S4a, b) [44, 45]. We additionally carried out mitochondrial pathway scores on these twelve types of neuronal cells and examined the pathway differences between APOE2/3 and APOE3/3 individuals. Our analysis indicates that excitatory pyramidal neurons (Ex-PYR) and parvalbumin-positive basket interneurons (In-PV(Basket)) are likely the primary cell types influenced by APOE2 in terms of mitochondrial function (Fig. S4c, Supplementary Table S11).

### APOE2 alters mitochondrial functions in Aβ1-42 -stimulated SH-SY5Y cells

**Fig. 3:**
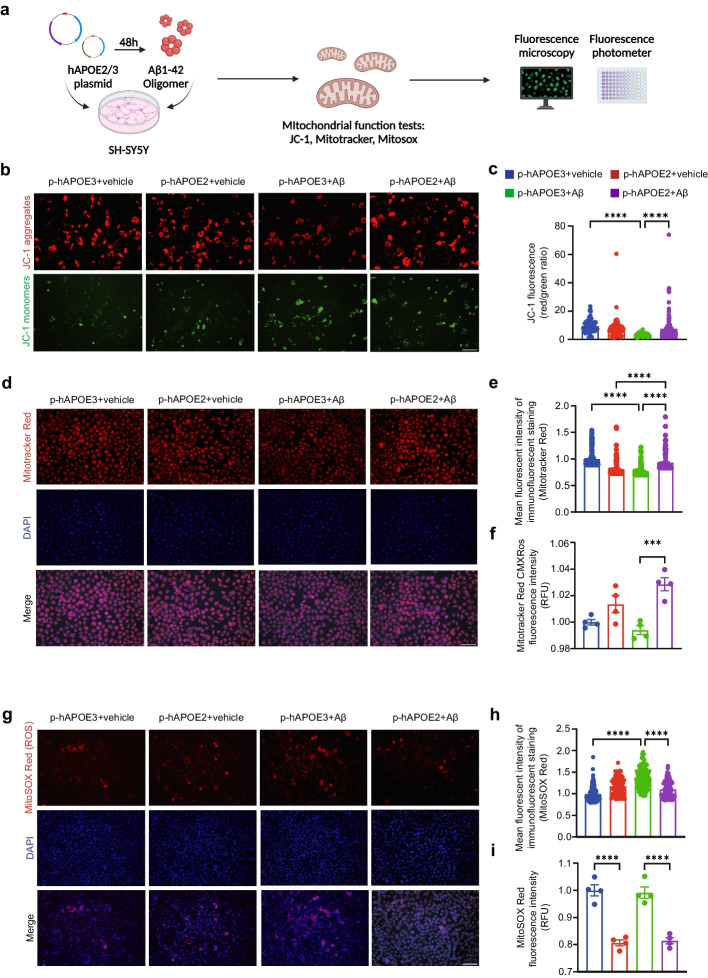
APOE2 alters mitochondrial functions in Aβ1-42 stimulated SH-SY5Y cells. **a **Experimental design for detecting mitochondrial functions of overexpressed APOE2 or APOE3 in Aβ1-42 or vehicle treated SH-SY5Y cells (Created with https://www.biorender.com). **b** Representative fluorescence images of JC-1in Aβ1-42 or vehicle treated SH-SY5Y cells. JC-1 aggregates (red) and monomers (green) distributions after loading with JC-1 (1 μg/ml). Scale bars, 100 μm. **c** Ratios of the fluorescence intensities of JC-1 labelling. JC-1 aggregates and JC-1 monomers were measured by average cell fluorescence intensity by fluorescence microscopy. **d** Representative fluorescence images of mitochondrial membrane potential (MMP) in SH-SY5Y cells using end-point assay. DAPI (blue) and MitoTracker (red). Scale bars, 100 μm. **e**, **f** Quantification of MMP using MitoTracker-Red fluorescent probes. The MMP was measured by average cell fluorescence intensity by fluorescence microscopy (**e**) and fluorescence light intensity tested by fluoresce microplate reader (**f**). **g**. Representative fluorescence images of mitochondrial levels of reactive oxygen species (ROS) in SH-SY5Y cells. DAPI (Blue) and MitoSox (Red). Scale bars, 100 μm. **h**, **i**. Quantification of mitochondrial ROS levels using MitoSox-Red fluorescent probes. The ROS level was measured by average cell fluorescence intensity by fluorescence microscopy (**h**) and fluorescence light intensity tested by fluoresce microplate reader (**i**). **c**, **e**, **h** Data are presented as the mean ± S.E. The experiment had three independent biological replicates (Kruskal-Wallis test). *P < 0.05; ***P < 0.001; ****P < 0.0001. **f**, **i** Data are presented as the mean ± S.E. The experiment had three independent biological replicates (One-way ANOVA). *P < 0.05; ***P < 0.001; ****P < 0.0001

To verify the protective effects of APOE2 on neuronal mitochondrion in AD pathology, we overexpress human APOE2 or APOE3 gene in mammalian SH-SY5Y cell line through plasmids and 48 h after transfection, SH-SY5Y cells were treated with Aβ1-42 oligomers (10 μM) for another 24 h. Mitochondrion tests including tests for end point of mitochondrial membrane potential, mitochondrial membrane potential dynamics, and mitochondrial superoxide production were conducted 24 h after Aβ1-42 oligomer incubation (Fig. 3a). We used JC-1 kit to investigate dynamic changes in mitochondrial membrane potential (MMP) ΔΨm and found that the MMP was significantly depolarized after Aβ1-42 oligomers incubation (Fig. 3b, c, *P*_p-hAPOE3+vehicle *vs* p-hAPOE3 +Aβo_ < 0.0001), which was restored in APOE2 groups (Fig. 3b, c, *P*_p-hAPOE2+Aβo *vs* p-hAPOE3 +Aβo_ < 0.0001). We also used MitoTracker Red CMXRos Kit to specifically label biologically active mitochondria and to measure the MMP in SH-SY5Y cells. According to the fluorescence imaging, MMP was significantly decreased in hAPOE3 groups after Aβ1-42 oligomers treatment (Fig. 3d, e, *P*_p-hAPOE3+vehicle *vs* p-hAPOE3+Aβo_ < 0.0001; *P*_p-hAPOE2+vehicle *vs* p-hAPOE2+Aβo_ < 0.0001), while we found elevated of MMP in APOE2 overexpressing cells compared to APOE3 overexpressing cells after Aβ1-42 oligomers treatment (*P*_p-hAPOE2+Aβo *vs* p-hAPOE3 +Aβo_ < 0.0001). Similarly, there was an increase in the MMP of APOE2 overexpressing cells after Aβ1-42 oligomers treatment by measuring fluorescence intensity (Fig. 3f, *P*_p-hAPOE2+Aβo *vs* p-hAPOE3 +Aβo_ = 0.0007). In addition, we examined the ROS level in the mitochondria using MitoSox-Red fluorescent probes. The fluorescence imaging showed that ROS accumulated in mitochondria after Aβ1-42 oligomers treatment (Fig. 3g, h, *P*_p-hAPOE3+vehicle *vs* p-hAPOE3+Aβo_ < 0.0001; *P*_p-hAPOE2+vehicle *vs* p-hAPOE2+Aβo_ = 0.0738), whereas APOE2 overexpression ameliorated ROS accumulation (Fig. 3g, h, *P*_p-hAPOE2+Aβo *vs* p-hAPOE3 +Aβo_ < 0.0001). Similar decrease in ROS level of APOE2 overexpressing cells after Aβ1-42 oligomers treatment was observed by measuring MitoSOX Red fluorescence intensity (Fig. 3i, *P*_p-hAPOE2+Aβo *vs* p-hAPOE3 +Aβo_ < 0.0001). Together, these results suggested that APOE2 could ameliorate Aβ-induced mitochondrial abnormalities in neuron-like cells.

### ApoE2 interacts with ERRα to influence neuronal mitochondrial functions

**Fig. 4:**
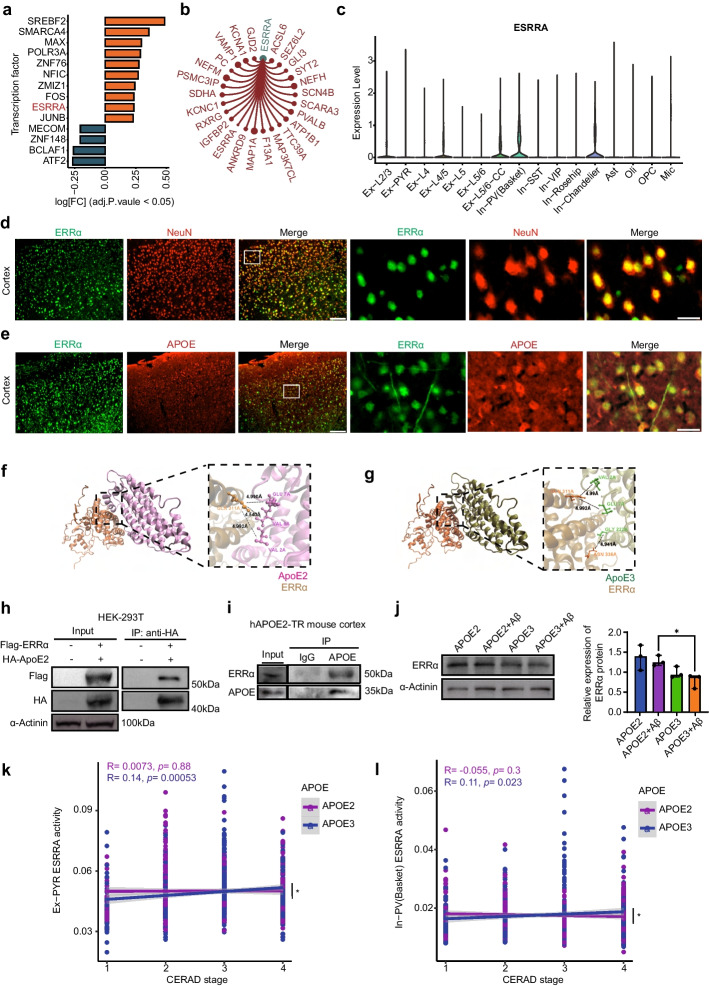
ApoE2 interacts with ERRα. **a** SCENIC analysis of different transcription factors (TFs) activity between APOE2 versus APOE3 neurons under AD pathology (adjusted. *P* value < 0.05). **b** The predicted target genes of ESRRA. **c** The distribution of ESRRA gene expression in dorsolateral prefrontal cortex (DLPFC). **d** Co-localization of the ERRα protein (green) with the neuronal marker NeuN (red) in mouse cortex. Scale bars, 100 μm for low magnification, 25 μm for high magnification. **e** Co-localization of the ERRα protein (green) with ApoE (red) in mouse cortex. Scale bars, 100 μm for low magnification, 25 μm for high magnification. **f**, **g** Binding mode of ApoE2 (**e**) or ApoE3 (**f**) and ERRα predicted by HDOCK. Left: overall structure of ApoE2 or ApoE3 bound to ERRα in cartoon view. ApoE2, ApoE3, and ERRα were colored in pink, dark green, and orange, respectively. Right: detailed interaction network between ApoE2 or ApoE3 and ERRα. Key residues of ApoE2 (pink) or ApoE3 (green) and ERRα (orange) were displayed as sticks. H-bonds are displayed in black dashed lines, and the distances (acceptor to donor heavy atom) of H-bonds are labeled. **h**, **i** Co-IP indicating the direct bind of ERRα protein and ApoE2 protein in HEK-293 T cells (**h**) and hAPOE2-TR mouse cortex (**i**). **j** Representative immunoblotting images of ERRα protein expression after APOE2 or APOE3 plasmid transfection and Aβ1-42 stimulation. Data were presented as the mean ± S.E.M. The experiment had three independent biological replicates (One-way ANOVA); *P < 0.05; **P < 0.01. **k** The correlation between ESRRA activity and CERAD pathology between APOE genotypes in Ex-PYR neurons (APOE2: *R =* 0.0073, *P* = 0.88; APOE3: *R =* 0.14, *P* = 0.00053). A significant difference between these two groups was confirmed through Cocor (*Z* = -2.1449, *P* = 0.0320); *P < 0.05. **l** The correlation between ESRRA activity and CERAD pathology between APOE genotypes in In-PV (Basket) neurons (APOE2: *R =* -0.053, *P* = 0.3; APOE3: *R =* 0.11, *P* = 0.023). A significant difference between these two groups was confirmed through Cocor (*Z* = -2.2957, *P* = 0.0217); *P < 0.05

To investigate the mechanism by which APOE2 influences neuronal mitochondrial functions, we used SCENIC to reconstruct gene regulatory networks and assessed the enrichment of transcription factors (TFs) and the activity of regulons [26]. We calculated the transcription factor activity of each neuron under AD pathology and utilized limma for differential analysis to screen out 14 transcription factors as potential APOE targets that exhibited differential activities in APOE2/3 and APOE3/3 neurons (Fig. 4a).

Among 14 candidates, ESRRA, which encodes estrogen-related receptor alpha (ERRα), has been reported to be central for mitochondrial biogenesis and ROS detection [46, 47]. Meanwhile, our analysis showed ESRRA regulated downstream molecules related to mitochondrial and neuronal functions (Fig. 4b) and was specifically highly expressed in human neurons (Fig. 4c). Immunofluorescent analysis proved co-localization of the ERRα protein with the neuronal marker NeuN (Fig. 4d). We found that under AD pathology, neuronal ESRRA expression significantly decreased (Fig.S5a). Even though there was no difference in ESRRA expression between males and females in the overall population (Fig.S5b), ESRRA expression in females significantly declined, whereas in males, it appeared to be unaffected under AD pathology (Fig.S4c–d).

Due to the co-localization of ERRα and ApoE in the cytoplasm of mouse cortical neurons (Fig. 4e), we hypothesized that ERRα might induce downstream effects through a direct interaction with ApoE. Molecular docking analysis indicated a possible interaction between ApoE2/ApoE3 and ERRα (Fig. 4f, g). The interaction between ApoE2 and ERRα was confirmed by co-IP assays both in HEK-293 T cells and in the cortex of hAPOE2-TR mice (Fig. 4h, i). We also found the binding between ERRα and ApoE3 in the cortex of hAPOE3-TR mice (Fig. S6a). Interestingly, ApoE2 overexpression can increase ERRα protein expression level in SH-SY5Y cells (Fig. 4j). Owing to the dropout characteristic of single-cell sequencing, we compensated for the missing expression values of ESRRA in neurons using the SAVER method [23]. Our analysis revealed a pronounced expression of ESRRA predominantly in Ex-PYR and In-PV(Basket) neurons. Notably, these neuron types are the most significantly impacted by APOE2 (Fig. S5d). This observation might suggest that the protective mechanism of APOE2 is potentially mediated through ESRRA. To elucidate differential influences exerted by ApoE2 and ApoE3 on ESRRA, we investigated the correlation between ESRRA activity and CERAD pathology in different APOE genotypes, particularly focusing on Ex-PYR and In-PV (Basket) neurons. The findings indicated a decline in ESRRA activity correlating with aggravated pathology in individuals with APOE3/3 genotype (Fig. 4k, l, Ex-PYR: *R =* 0.14, *P* = 0.00053; In-PV (Basket): *R =* 0.11, *P* = 0.023). Conversely, ESRRA activity levels were relatively stable in APOE2/3 individuals (Fig. 4k, l, Ex-PYR: *R =* 0.0073, *P* = 0.88; In-PV (Basket): *R = -*0.053, *P* = 0.3). A significant difference between these two groups was confirmed using the R package Cocor (Fig. 4j, k, Ex-PYR: Z = -2.1449, *P* = 0.0320; In-PV (Basket): Z = -2.2957, *P* = 0.0217). These results imply the potential disparities in the effects of ApoE2 and ApoE3 on ERRα.

Therefore, ApoE2 interacted with ERRα and then regulated mitochondria-related molecules, especially in neurons.

### Elevated ERRα expression restores mitochondrial function in Aβ1-42 stimulated SH-SY5Y cells

To figure out the role of ERRα mediated ApoE2 protective effect, we used human ESRRA overexpression plasmids or ERRα agonist to verify the influence of ApoE2-ERRα signaling on mitochondrial function in Aβ1-42 stimulated SH-SY5Y cells.

**Fig. 5:**
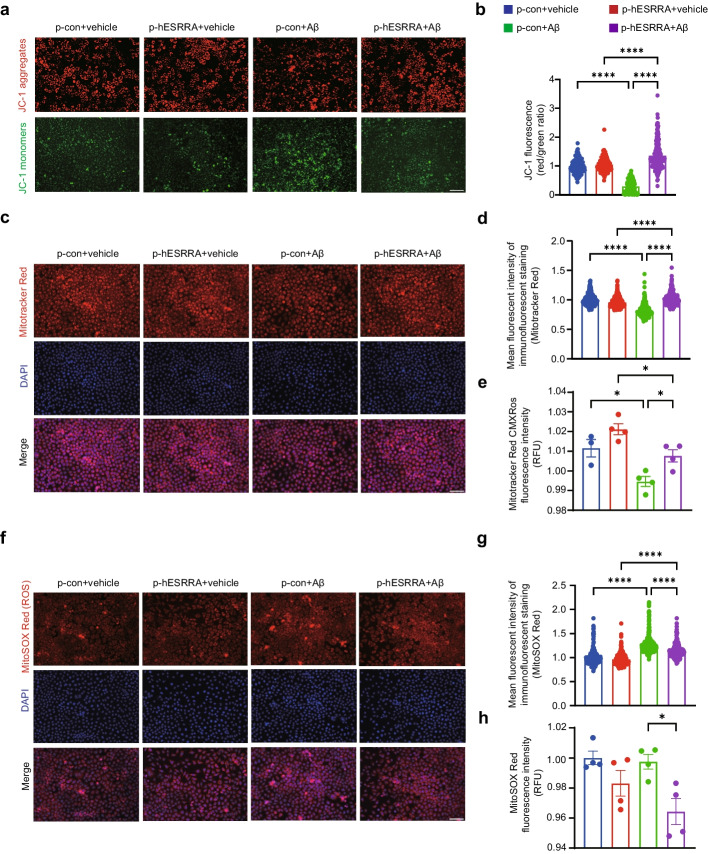
ESRRA overexpression affects mitochondrial function in Aβ1-42-stimulated SH-SY5Y cells. **a** Representative fluorescence images of JC-1 in SH-SY5Y cells. JC-1 aggregates (red) and monomers (green) distributions after loading with JC-1 (1 μg/ml) probes. Scale bars, 100 μm. **b** Ratios of the fluorescence intensities of JC-1 labelling. JC-1 aggregates and JC-1 monomers were measured by average cell fluorescence intensity by fluorescence microscopy. **c** Representative fluorescence images of MMP in SH-SY5Y cells. DAPI (blue) and MitoTracker (red). Scale bars, 100 μm. **d**, **e** Quantification of MMP using MitoTracker-Red fluorescent probes. The MMP was measured by average cell fluorescence intensity by fluorescence microscopy (**d**) and fluorescence light intensity tested by fluoresce microplate reader (**e**). **f** Representative fluorescence images of mitochondrial levels of ROS in SH-SY5Y cells. DAPI (blue) and MitoSox (red). Scale bars, 100 μm. **g**, **h** Quantification of mitochondrial ROS levels using MitoSox-Red fluorescent probes. The ROS level was measured by average cell fluorescence intensity by fluorescence microscopy (**g**) and fluorescence light intensity tested by fluoresce microplate reader (**h**). **e**, **h** Data are presented as the mean ± S.E. The experiment had three independent biological replicates (Kruskal-Wallis test). *P < 0.05; ***P < 0.001; ****P < 0.0001. **b**, **d**, **g** Data were presented as the mean ± S.E. The experiment had three independent biological replicates (One-way ANOVA); *P < 0.05; ****P < 0.0001

Firstly, we investigated whether overexpression of human ESRRA could affect mitochondrial function in Aβ1-42 treated SH-SY5Y cells by detecting mitochondrial membrane potential ΔΨm using JC-1 kit. We found that the ΔΨm was depolarized after Aβ1-42 oligomers incubation, which was reversed by ESRRA overexpression (Fig. 5a, b, *P*_p-con+vehicle* vs* p-con+Aβo_ < 0.0001; *P*_p-hESRRA+vehicle *vs* p-hESRRA+Aβo_ < 0.0001; *P*_p-con+Aβo *vs* p-hESRRA+Aβo_ < 0.0001). We also used MitoTracker Red CMXRos Kit to detect MMP in SH-SY5Y cells. According to the fluorescence imaging, MMP was significantly decreased by Aβ1-42 oligomers in control groups (Fig. 5c, d, *P*_p-con+vehicle* vs* p-con+Aβo_ < 0.0001; *P*_p-hESRRA+vehicle *vs* p-hESRRA+Aβo_ < 0.0001) and significantly increased by ESRRA overexpression (Fig. 5c, d, *P*_p-con+Aβo *vs* p-hESRRA +Aβo_ < 0.0001). We also observed a similar decrease of MMP in Aβ1-42 oligomers treated cells (Fig. 5e, *P*_p-con+vehicle* vs* p-con+Aβo_ = 0.0178; *P*_p-hESRRA+vehicle *vs* p-hESRRA+Aβo_ = 0.0416) and that was increased in ESRRA overexpressing cells after Aβ1-42 oligomers treatment (Fig. 5e, *P*_p-hESRRA+ vehicle vs p-hESRRA +Aβo_ = 0.0490) according to the fluorescence intensity. Additionally, we measured the ROS level in SY5Y cells using MitoSox-Red fluorescent probes. The fluorescence imaging showed that ROS accumulated in mitochondria after Aβ1-42 oligomers treatment (Fig. 5f, g, *P*_p-con+vehicle* vs* p-con+Aβo_ < 0.0001; *P*_p-hESRRA+vehicle *vs* p-hESRRA+Aβo_ < 0.0001), whereas ESRRA overexpression ameliorated ROS accumulation (Fig. 5f, g, *P*_p-con+Aβo* vs* p-hESRRA+Aβo_ < 0.0001). Similarly, decrease ROS level was observed in ESRRA overexpressing cells after Aβ1-42 oligomers treatment by measuring MitoSOX Red fluorescence intensity (Fig. 5h, *P*_p-con+Aβo* vs* p-hESRRA+Aβo_ = 0.0191). Therefore, overexpressed human ESRRA showed protective effects on mitochondrial function in Aβ1-42 oligomers treated SH-SY5Y cells, which was similar to APOE2 overexpression.

**Fig. 6:**
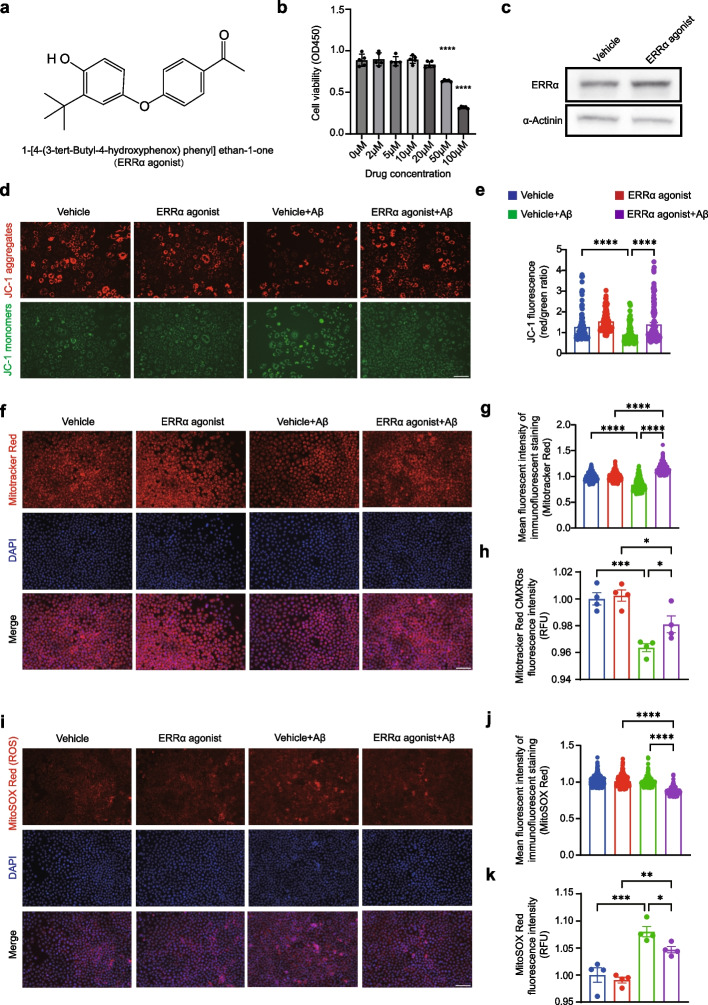
ERRα agonist alters mitochondrial functions in Aβ1-42 stimulated SH-SY5Y cells. **a** The structural formula of 1-[4-(3-tert-Butyl-4-hydroxyphenox) phenyl] ethan-1-one (ERRα agonist). **b** Cell viability of SH-SY5Y cells treated with ERRα agonist at indicated concentrations was determined by CCK8 assay. **c** Representative immunoblotting images of ERRα protein expression after agonist treatment in SH-SY5Y cells. **d** Representative fluorescence images of JC-1. JC-1 aggregates (red) and monomers (green) distributions after loading with JC-1 (1 μg/ml). Scale bars, 100 μm. **e** Ratios of the fluorescence intensities of JC-1 labelling. JC-1 aggregates and JC-1 monomers were measured by average cell fluorescence intensity by fluorescence microscopy. **f** Representative fluorescence images of end-point MMP in SH-SY5Y cells. DAPI (blue) and MitoTracker (red). Scale bars, 100 μm. **g**, **h** Quantification of MMP using MitoTracker-Red fluorescent probes. The MMP was measured by average cell fluorescence intensity by fluorescence microscopy (**g**) and fluorescence light intensity tested by fluoresce microplate reader (**h**). **i** Representative fluorescence images of mitochondrial ROS levels in SH-SY5Y cells. DAPI (blue) and MitoSox (red). Scale bars, 100 μm. **j**, **k** Quantification of mitochondrial ROS levels using MitoSox-Red fluorescent probes. The ROS level was measured by average cell fluorescence intensity by fluorescence and fluorescence light intensity tested by fluoresce microplate reader. **e**, **g**, **j** Data were presented as the mean ± S.E. The experiment had three independent biological replicates (Kruskal–Wallis test); *P < 0.05; **P < 0.01; ***P < 0.001; ****P < 0.0001. **b**, **h**, **k** Data were presented as the mean ± S.E. The experiment had three independent biological replicates (One-way ANOVA); *P < 0.05; **P < 0.01; ***P < 0.001; ****P < 0.0001

We then used 1-[4-(3-tert-Butyl-4-hydroxyphenox) phenyl] ethan-1-one (Fig. 6a), an ERRα agonist, to elevate the function of ERRα [33]. After treated with ERRα agonist at 0–20 μM, SH-SY5Y cells showed no significant change in cell viability (Fig. 6b). The expression level of ERRα was increased after the ERRα agonist treatment (Fig. 6c). Therefore, the safety and effectiveness of the ERRα agonist were validated. Then we also test the mitochondrial function of Aβ1-42 oligomers treated SH-SY5Y cells after ERRα agonist treatment (5 μM). Similar to overexpression of ESRRA, the depolarized MMP ΔΨm was reversed by ERRα agonist in SH-SY5Y cells after Aβ1-42 oligomers incubation (Fig. 6d, e, *P*_vehicle+Aβo *vs* ERRα agonist+Aβo_ < 0.0001). We also observed increased MMP (Fig. 6f, g, *P*_vehicle+Aβo *vs* ERRα agonist+Aβo_ < 0.0001; Fig. 6h, *P*_vehicle+Aβo *vs* ERRα agonist+Aβo_ = 0.0409) and reduced ROS (Fig. 6i, j, *P*_vehicle+Aβo *vs* ERRα agonist+Aβo_ < 0.0001; Fig. 6k, *P*_vehicle+Aβo *vs* ERRα agonist+Aβo_ = 0.0466) after ERRα agonist treatment. Together, these results indicate ERRα activation ameliorate Aβ-induced mitochondrial abnormalities in neuron-like cells.

### ERRα agonist ameliorates cognitive deficits in AD model mice

**Fig. 7:**
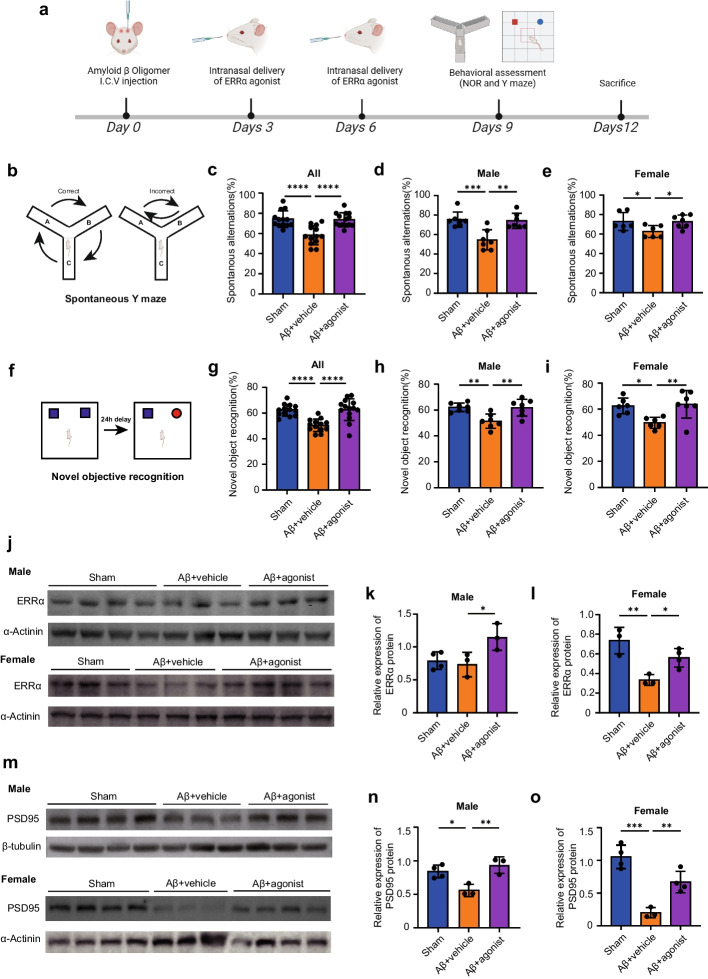
ERRα agonist treatment ameliorate cognitive function in Aβ1-42 ICV injected mice. **a** Experimental design for Aβ1-42 injection and behavioral test (Created with https://www.biorender.com). **b** Schematic representation of Y-maze task. **c**–**e** The spontaneous alternation rates of all mice (**c**, *n =* 13, sham group; *n =* 13, Aβ + vehicle group; *n =* 14, Aβ + agonist group), male mice (**d**, *n =* 7, sham group; *n =* 7, Aβ + vehicle group; *n =* 7, Aβ + agonist group), and female mice (**e**, *n =* 6, sham group; *n =* 6, Aβ + vehicle group; *n =* 7, Aβ + agonist group) in Y-maze were showed. **f** Schematic representation of NOR task. **g**–**i** The novel objective recognition rates of all mice (**g,** *n =* 13, sham group; *n =* 13, Aβ + vehicle group; *n =* 14, Aβ + agonist group) male mice (**h,** *n =* 7, sham group; *n =* 7, Aβ + vehicle group; *n =* 7, Aβ + agonist group), and female mice (**i,** *n =* 6, sham group; *n =* 6, Aβ + vehicle group; *n =* 7, Aβ + agonist group) were showed. **j**. Representative immunoblotting images of ERRα protein expression from cortex, with the top image representing males and the bottom image representing females. **k**, **l** Quantification analysis of cortex ERRα protein expression in male mice (**k,** *n =* 4, sham group; *n =* 3, Aβ + vehicle group; *n =* 3, Aβ + agonist group) and female mice (**l,** *n =* 3, sham group; *n =* 3, Aβ + vehicle group; *n =* 4, Aβ + agonist group). **m** Representative immunoblotting images of PSD95 protein expression from cortex, with the top image representing males and the bottom image representing females. **n**, **o** Quantification analysis of cortex PSD95 protein expression in male mice (**n** *n =* 4, sham group; *n =* 3, Aβ + vehicle group; *n =* 3, Aβ + agonist group) and female mice (**o,** *n =* 4, sham group; *n =* 3, Aβ + vehicle group; *n =* 4, Aβ + agonist group). **c**, **d**, **e**, **g**, **h**, **i**, **k**, **l**, **n**, **o** Data were presented as the mean ± S.E; One-way ANOVA; *P < 0.05; **P < 0.01; ***P < 0.001; ****P < 0.0001

To figure out whether ERRα agonist rescue the cognitive decline in AD mice. We established an Aβ-induced AD model by intracerebroventricular (ICV) injection of Aβ oligomer (400 pmol/mouse) in ICR mice [34]. Then, the Aβ-induced AD mice were treated with ERRα agonist (1 mg/kg) or vehicle intranasally twice, on the third and sixth days after induction (Fig. 7a). Y maze and novel object recognition test were performed on the ninth day after Aβ injection to assess the cognitive performance (Fig. 7a). Considering the potential sex differences associated with ERRα, we included an equal number of male and female mice in our study. The Aβ-induced AD mice showed lower spontaneous alternations in Y maze test (Fig. 7b, c), while ERRα agonist treatment improve the Y maze performance of mice compared to the vehicle-treated group (Fig. 7b, c), with similar changes observed in both male and female mice (Fig. 7d, e). Moreover, the loss performance for novel object of Aβ-induced AD mice were restored by ERRα agonist in novel object recognition test (Fig. 7f, g). Similarly, this improvement was seen in both male and female mice (Fig. 7h, i). We discovered that Aβ caused a decrease in ERRα protein levels in the cortex of female mice without significant changes in males (Fig. 7j–l), consistent with previous human brain ESRRA expression analyses (Fig. S5d), indicating a more noticeable decrease in females affected by AD pathology. Regardless of changes in ERRα protein expression, activating ERRα enhanced its expression in the cortex, offering protective effects (Fig. 7j–l). Intranasal administration, allowing delivery to different brain regions [48], ERRα immunofluorescent staining of male mice showed significant ERRα positive cell number increase in the perirhinal cortex and hippocampal dentate gyrus region, but not in the CA1 area, suggesting the agonist’s effectiveness might be mediated through effects on the cortex and hippocampus (Fig. S7a–f). Post-treatment with the ERRα agonist, we observed significant recovery of PSD95 levels in the cortex of Aβ-induced mice (Fig. 7m–o), while cortex synaptophysin and Homer1 levels showed no significant changes (Fig. S7g–j). Additionally, an increase in PSD95 expression was observed in the hippocampus post ERRα agonist administration (Fig. S7k, l). Therefore, the improved cognitive performance in ERRα agonist treated mice might derive from the upregulation of PSD95.

## Discussion

Human APOE isoforms have been shown to influence the relative risk of late-onset AD. While many studies mainly focused on the role of APOE4 in AD, little is known about how APOE2 correlates with AD, despite APOE2 being widely acknowledged for its neuroprotective properties. The mechanisms underlying APOE2's neuroprotection are not yet fully understood [49]. Findings from the present study uncovered the correlation between APOE2 and neuronal mitochondria. Examination of mitochondrial function revealed the protective effects of APOE2 such as restored the depolarized ΔΨm, increased MMP, and reduced ROS in Aβ damaged neuron-like cells. Notably, we revealed for the first time ApoE2 interaction with ERRα showed protective effects on the neuronal mitochondrial. ERRα overexpression and activation in Aβ induced neuron-like cells was sufficient for rescuing mitochondrial dysfunction, and ERRα agonist restored cognitive deficit in Aβ-induced AD mice. Together, the findings identify APOE2-ERRα-dependent regulation of mitochondrial function as a mechanism during AD pathology.

It is well known that “Aβ cascade hypothesis” may be the key mechanism in AD [50, 51]. The hypothesis proposes that the primary and pivotal event in the development of AD is the buildup of Aβ. Emerging evidence suggests a strong connection between mitochondrial dysfunction and AD pathology, potentially playing a synergistic role [52]. The ‘Mitochondrial Cascade Hypothesis’ suggests that mitochondrial dysfunction is a vital mechanism of AD pathology, and is an incipient event in the progression of AD [53–56]. Mitochondria are intensely dynamic and complex organelles critical for energy production, and also play a significant role in processes such as cell death, signaling pathways, apoptosis, ROS generation, and calcium homeostasis, which are disrupted in neurodegenerative disorders. Mitochondrial dysfunction damages the function of neurons, which leads to neuronal loss in neurodegenerative diseases. The presence of Aβ exacerbates mitochondrial dysfunction, and interventions at this stage might have significant effects, suggesting that secondary mitochondrial cascade responses may be key in AD. Our study provides some validation that therapeutic strategies based on the ‘Mitochondrial Cascade Hypothesis’ may hold considerable potential for improving AD outcomes. Further researches are needed to explore and distinguish the potential mechanisms in the future.

Previous studies have suggested that ApoE and its protein-digesting fragments [57–59] potentially target to mitochondria-associated pathways. ApoE (amino acids 1–299) can be cleaved by specific proteases in neurons, leading to the formation of carboxy-terminally truncated ApoE fragments (1–272) [59–61], which could disrupt function of neuronal mitochondria. ApoE4 interacts with mitochondria in many ways, including the capability of the ApoE4 fragment to locate within mitochondria [57], to bind with the subunits of the mitochondrial respiratory complexes III, IV, and V, disrupting mitochondrial activity [59], and even to interfere with the expression of mitochondrial transcription factors [55, 62, 63]. In our findings, APOE isoforms had a significant impact on the function of neuronal mitochondria. APOE2 might mitigate Aβ-induced mitochondrial dysfunction, thereby reducing the risk of AD. Our findings suggested that genetic factors influencing mitochondrial function might play a crucial role in the pathogenesis of AD. Although Aβ plaque buildup occurs in the pre-symptomatic phase of AD, the progression appears to decelerate over time; changes in bioenergetic metabolism could influence subsequent progression of Aβ levels and other pathological indicators [64]. Pathologies such as Aβ and tau may interact with mitochondria, thereby contributing to further disease progression. Therefore, the progression of AD and the accumulation of neurological deficits may not arise from a single linear cascade reaction, but rather from a complex interaction of multiple factors.

ERRα, also called ESRRA, is a type of nuclear receptor that has DNA sequence homology to estrogen receptor alpha (ESR1/ERα) [65]. Estrogen-related receptors (ERRs) crosstalk with estrogen signaling via transcriptional regulation or specific binding to co-response elements of target genes. The physiological and metabolic effects of ERRα have only recently attracted attention and are not yet fully understood. Several studies reveal that ERRα can regulate mitochondrial function, turnover, and lipolysis [66–70]. Besides, the protein expression level of ERRα exhibited a significant decrease in the cortex of 6 months APP/PS1 mice, pointing to the dysregulation of ERRα in AD [71]. Additionally, ESRRA knockout mice have been observed to display abnormal diet and social behaviors [72–74]. Here, our study found that APOE isoforms interacted with ERRα and overexpressed APOE2 increased the expression of ERRα. Findings in our study indicated ERRα may serve as a potential target for the prevention and treatment of AD. Further investigation is required to delineate the mechanism of different estrogen-related receptor subtypes and their effects on AD and aging individual.

Since then, no endogenous ligands for ESRRA have been identified except cholesterol [65, 75]. APOE is mainly expressed in the cytoplasm of neurons [75], while ERRα mainly functions in the nucleus [64]. Our immunofluorescence staining showed that they co-localized in the perinuclear cytoplasm of neurons of mice brain, which indicated that APOE might stimulate ERRα to enter the nucleus to act as a transcription factor and to promote downstream genes, thus regulating mitochondrial functions. However, our study did not delve into whether there are differences in the binding of various APOE isoforms to the estrogen-related receptor, and the specific molecular mechanisms by which APOE isoforms regulating the estrogen receptor (e.g., whether it involves the full-length ApoE protein or a cleaved fragment) remain unclear. Future research should figure out the molecular mechanism on APOE and estrogen-related receptors in AD pathology.

In our study, activation of ERRα has been shown to improve cognitive functions in the Aβ ICV injected mouse model. This model induces cognitive decline within a relatively short time, which is recognized for mimicking the sporadic form of AD during its early stages [76]. Different research found Aβ injection led to the decrease of PSD95, also observed in transgenic AD mouse models and among AD patients [77–79], underscoring its relevance in the AD pathology. Given that interventions of increasing PSD95 levels have led to cognitive improvements [80], our findings suggested that stimulating neuronal ERRα could potentially improve Aβ-induced cognitive deficits by reversing PSD95 decrease. However, the specific mechanisms remain to be fully elucidated. Future studies should use electrophysiology or other techniques to gain detailed insights into the relationship between ERRα and synaptic structure and functions. Furthermore, the efficacy of ERRα agonists warrants investigation in chronic AD models, including AD transgenic mice, to comprehensively evaluate ERRα's therapeutic potential for AD.

In conclusion, our investigation revealed that intervention in mitochondrial function mitigates metabolic abnormalities and oxidative damage in AD neurons. Further exploration is necessitated to decipher the underlying mechanisms of AD pathology, providing deeper insights into mitochondrial dysfunction, thereby facilitating the development of efficacious therapeutic interventions. Moreover, the protective influence exerted by the APOE2 gene may be correlated with enhanced mitochondrial function. This enhancement could be related to either direct or indirect interactions between APOE isoforms and estrogen-related receptors. Unraveling the specific mechanism of APOE2’s protective effects through biosignature analysis could potentially yield novel targets for AD treatment.

### Supplementary Information

Additional file 1.

Additional file 2.

## Data Availability

The single-nucleus RNA sequencing (snRNA-Seq) data can be accessed via https://www.synapse.org/#!Synapse:syn31512863 (ID: syn31512863) and https://www.synapse.org/#!Synapse: syn21261143 (ID: syn21261143). The bulk RNA-sequencing data can be accessed via https://www.synapse.org/#!Synapse:syn3388564 (ID: syn3388564).

## Abbreviations

AD: Alzheimer’s disease
APOE: Apolipoprotein E
Aβ: Beta-amyloid
CCCP: Carbonyl cyanide 3-chlorophenylhydrazone
Co-IP: Co-immunoprecipitation
DEGs: Differentially expressed genes
DLPFC: Dorsolateral prefrontal cortex
ER: Endoplasmic reticulum
ERRs: Estrogen-related receptors
ERRα: Estrogen-related receptor alpha
GO: Gene ontology
GSVA: Gene Set Variation Analysis
ICV: Intracerebroventricular
MMP: Mitochondrial membrane potential
PFC: Prefrontal cortex
ROSMAP: The Religious Orders Study and Memory and Aging Project
snRNA-Seq: Single-nucleus RNA sequencing
TFs: Transcription factors
TNF: Tumor necrosis factor
UMAP: Uniform manifold approximation and projection
ΔΨm: Mitochondrial transmembrane potential
