# Supplementary material for: APOE2 protects against Aβ pathology by improving neuronal mitochondrial function through ERRα signaling
Source: Cell Mol Biol Lett. 2024 Jun 12;29:87. doi: 10.1186/s11658-024-00600-x (PMC11170814; doi:10.1186/s11658-024-00600-x)
Supplement: Supplementary file 1 — Additional file 1. [file 11658_2024_600_MOESM1_ESM.docx]

**APOE2 protects against Aβ pathology by improving neuronal mitochondrial function through ERRα signaling**

Zhiyuan Ning, Ying Liu, Mengyao Wan, You Zuo, Siqi Chen, Zhongshan Shi, Yongteng Xu, Honghong Li, Ho Ko, Jing Zhang, Songhua Xiao, Daji Guo, Yamei Tang

**Supplementary Figure**


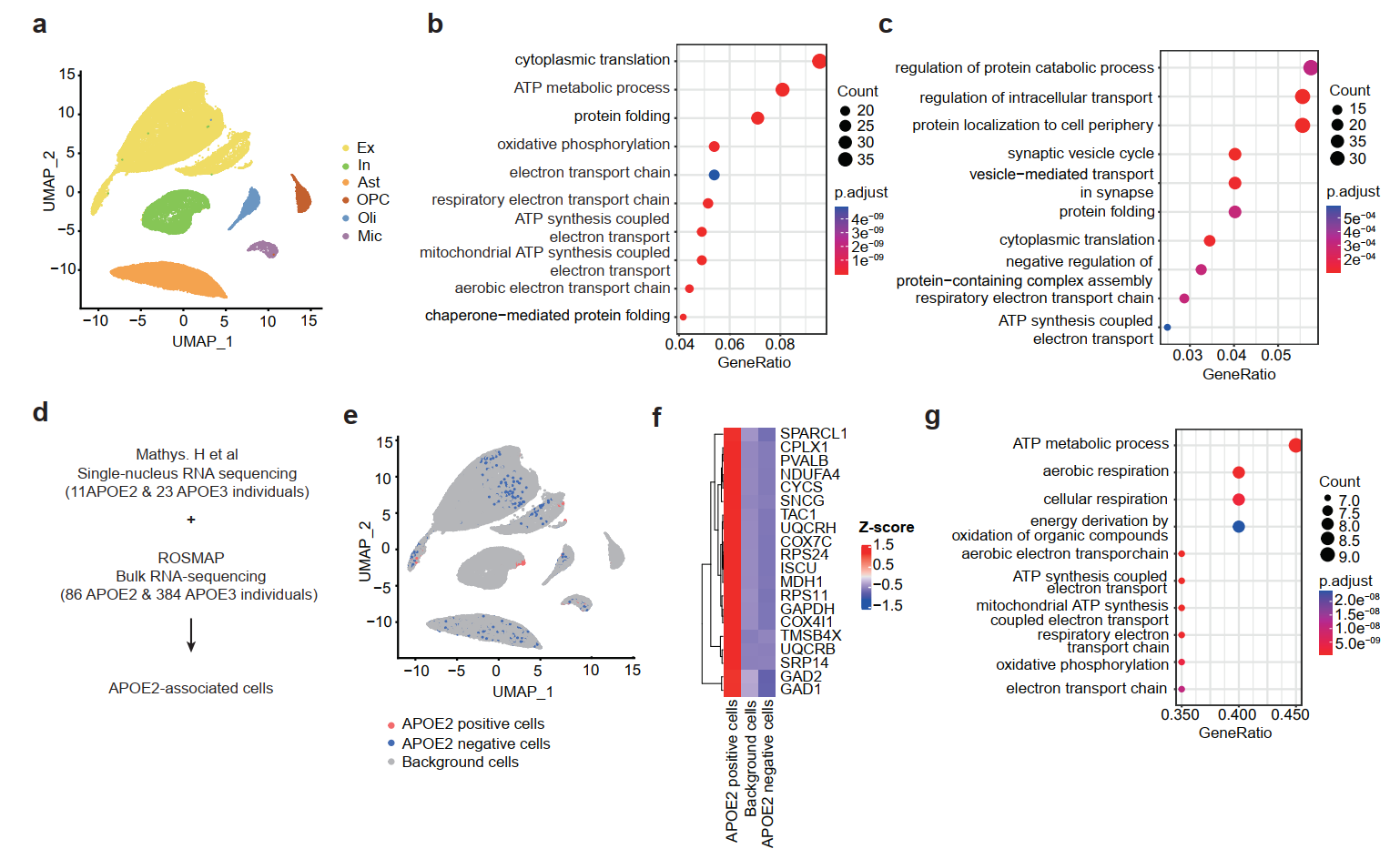


**Fig S1： Mathys.H et al single-nucleus RNA sequencing analysis**

1. The UMAP plot displayed six main cell types in Mathys.H et al single-nucleus RNA sequencing.
2. GO pathway enrichment of upregulated genes in APOE2 excitatory neurons compared to APOE3.
3. GO pathway enrichment of upregulated genes in APOE2 inhibitory neurons compared to APOE3.
4. Workflow of Scissor for identification of APOE2-associated cells.
5. The UMAP plot displayed APOE2 positive cells, APOE2 negative cells, and background cells.
6. Heatmap showing the top marker genes of APOE2 positive cells.
7. GO pathway enrichment of the top marker genes of APOE2 positive cells.


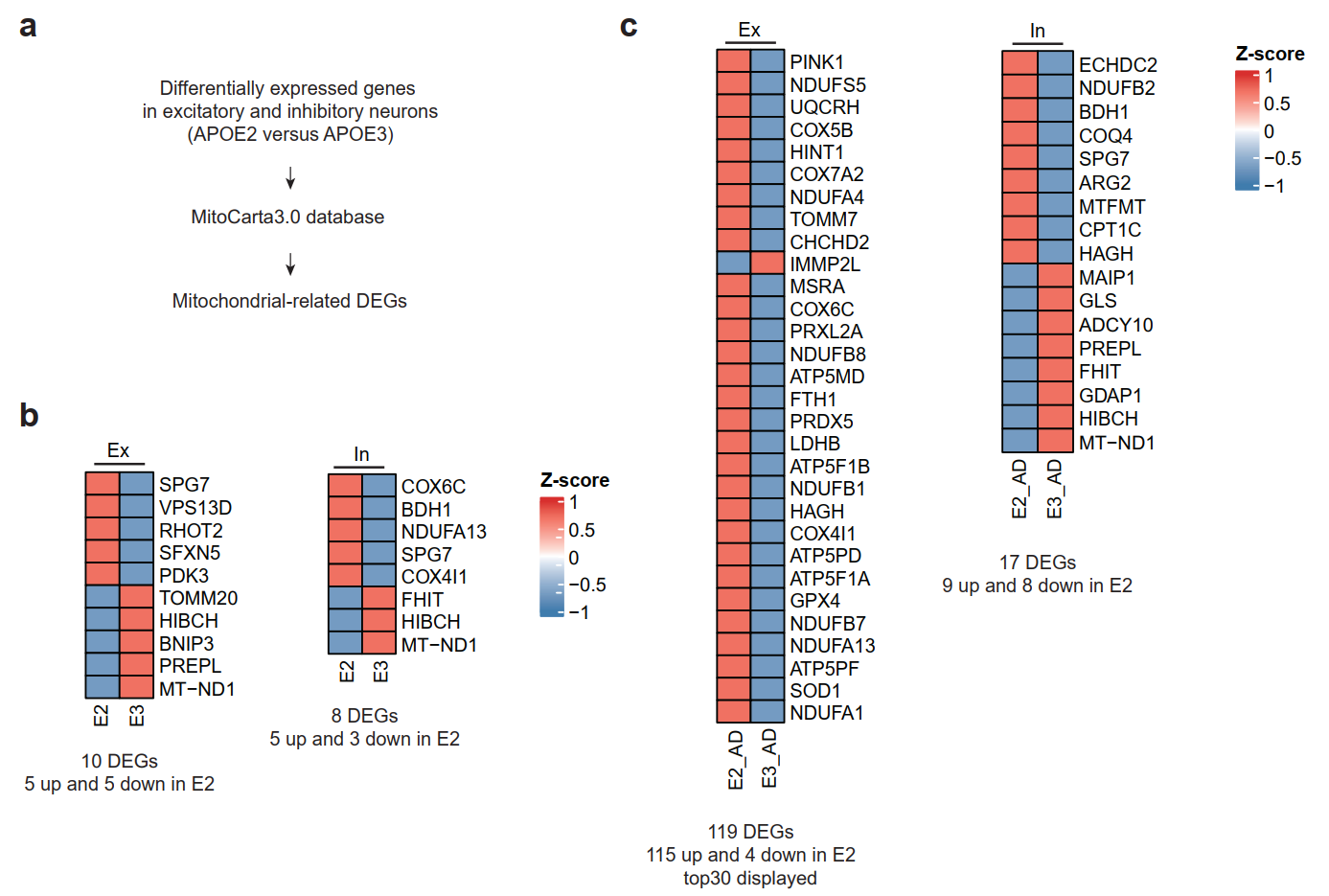


**Fig. S2：****Mitochondrial-related DEGs in APOE2 neurons versus APOE3 neurons**

1. Workflow of identification of Mitochondrial-related DEGs.
2. Mitochondrial-related DEGs in excitatory and inhibitory neurons. The color scale represented Z-scores.
3. Mitochondrial-related DEGs in excitatory and inhibitory neurons in AD pathology. The color scale represented Z-scores.


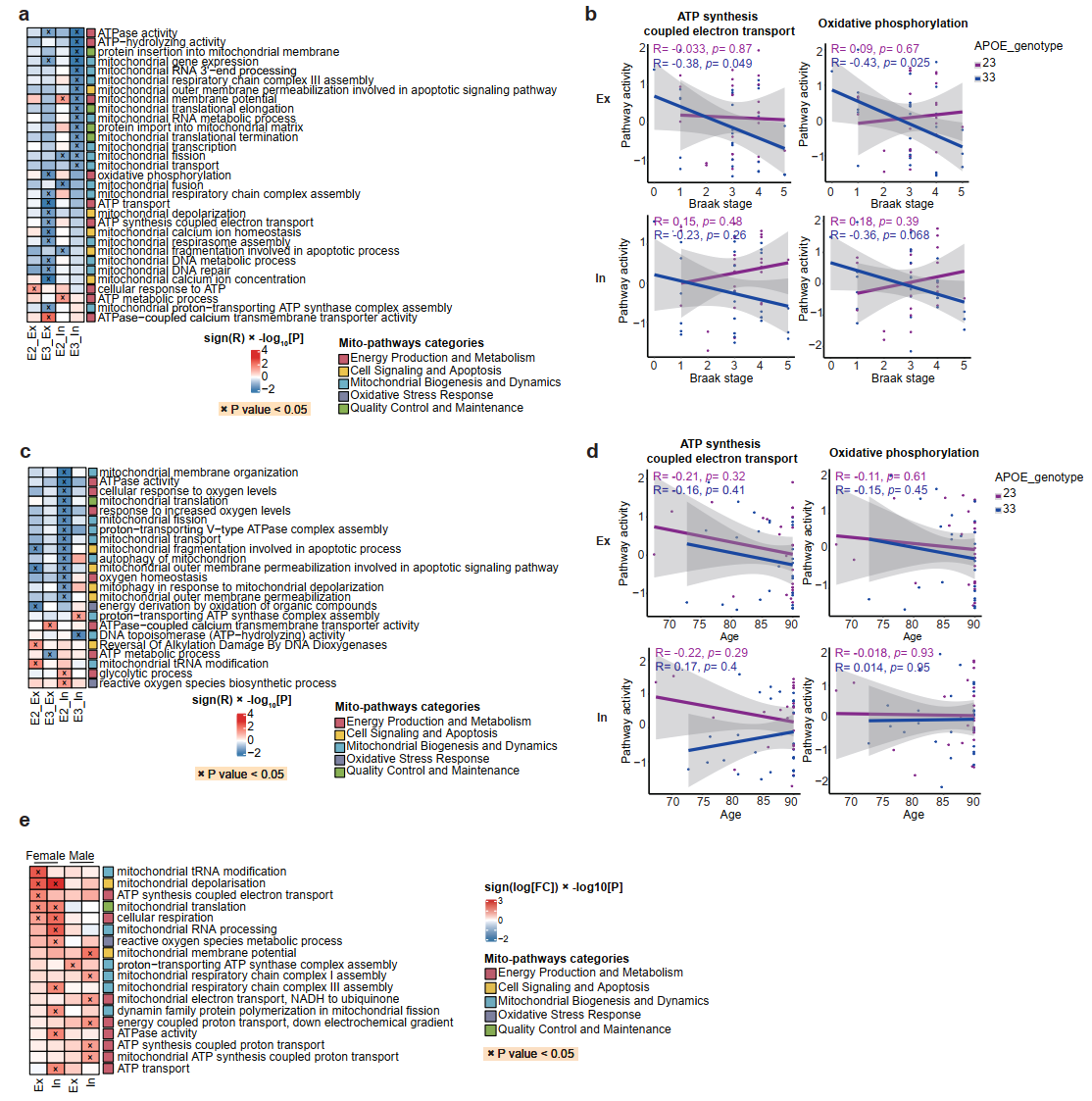


**Fig. S3:** **The influence of Braak stages, age, and gender on the impact of APOE2 on mitochondrial function**

1. The heatmap showed correlations between mitochondrial-related pathway scores and the Braak stages in APOE2 and APOE3 neurons. Red indicated positive correlation with the Braak stages and blue indicated negative correlation with the Braak stages. The color scale represented sign (R) ×log10[P] values. P < 0.05 indicated (x).
2. The scatter plots showed the pathway activity of ‘ATP synthesis coupled electron transport’ and ‘oxidative phosphorylation’ in APOE2 and APOE3 excitatory and inhibitory neurons with the progression of Braak stages.
3. The heatmap showed correlations between mitochondrial-related pathway scores and age in APOE2 and APOE3 neurons. Red indicated positive correlation with age and blue indicated negative correlation with age. The color scale represented sign (R) ×log10[P] values. P < 0.05 indicated (x).
4. The scatter plots showed the pathway activity of ‘ATP synthesis coupled electron transport’ and ‘oxidative phosphorylation’ in APOE2 and APOE3 excitatory and inhibitory neurons with age.
5. Heatmap showed mitochondrion-associated pathways altered in excitatory and inhibitory neurons in APOE2 versus APOE3 individuals stratified by gender. Red indicated APOE2 upregulation and blue indicated APOE2 downregulation. The color scale represents sign (log [FC]) ×log10[P] values. P < 0.05 indicated (x).


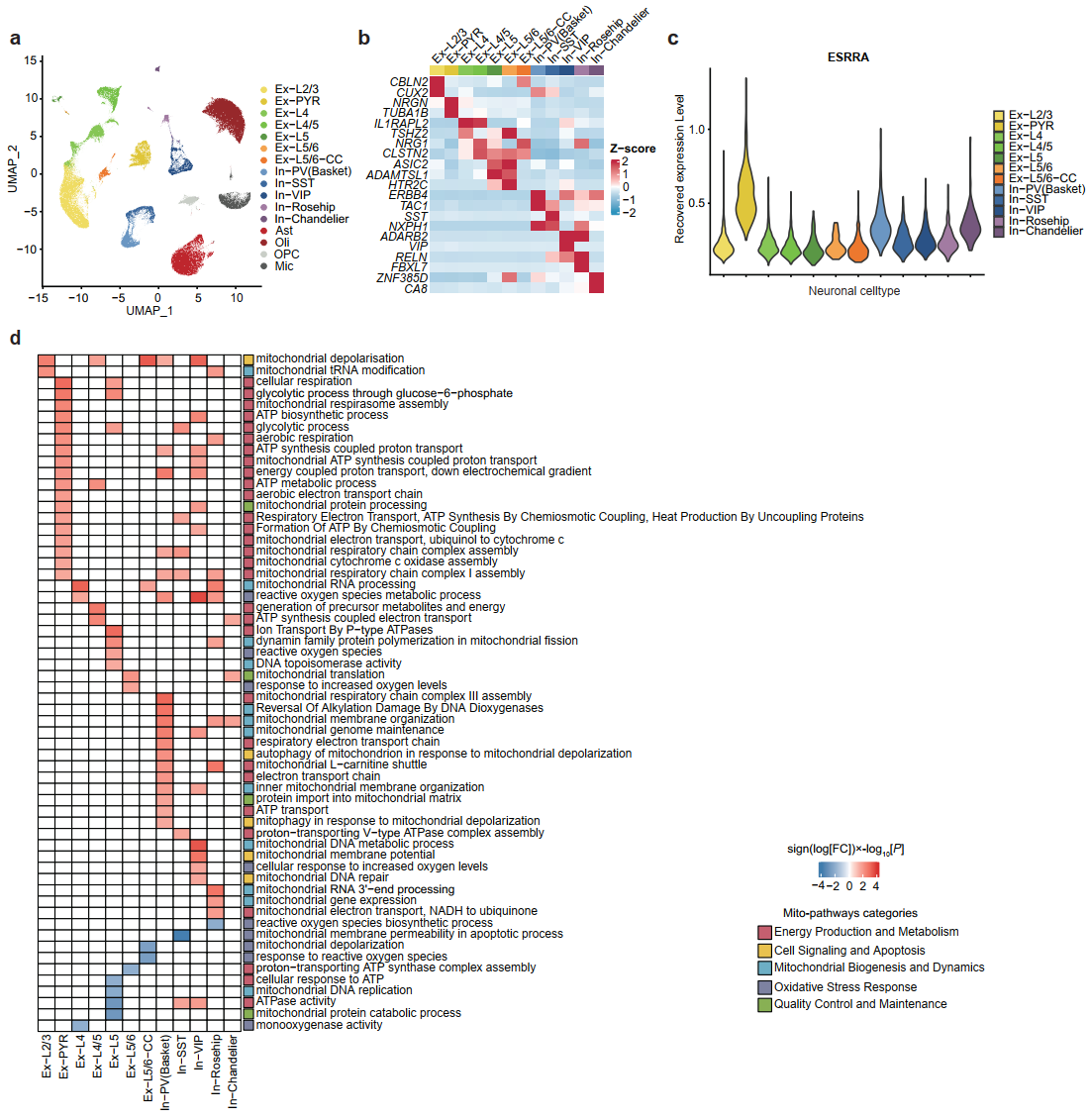


**Fig. S4: Excitatory pyramidal neurons (Ex-PYR) and parvalbumin-positive basket interneurons (In−PV(Basket)) are likely the primary cell types influenced by APOE2**

1. The UMAP plot displayed sixteen cell types in DLPFC, including seven cell types of excitatory neurons and five cell types of inhibitory neurons.
2. Heatmap showed the expression of representative marker genes in each neuronal cell type.
3. The distribution of recovered ESRRA gene expression in neuronal cell types.
4. Heatmap showed mitochondrion-associated pathways altered across major twelve neuronal cell types in APOE2 versus APOE3 individual (nominal P < 0.05, linear model). Red indicated APOE2 upregulation and blue indicated APOE2 downregulation. The color scale represents sign (log [FC]) ×log10[P] values. Pathways with absolute value of sign (log [FC]) ×log10[P]>1.3 were shown in the heatmap.

**
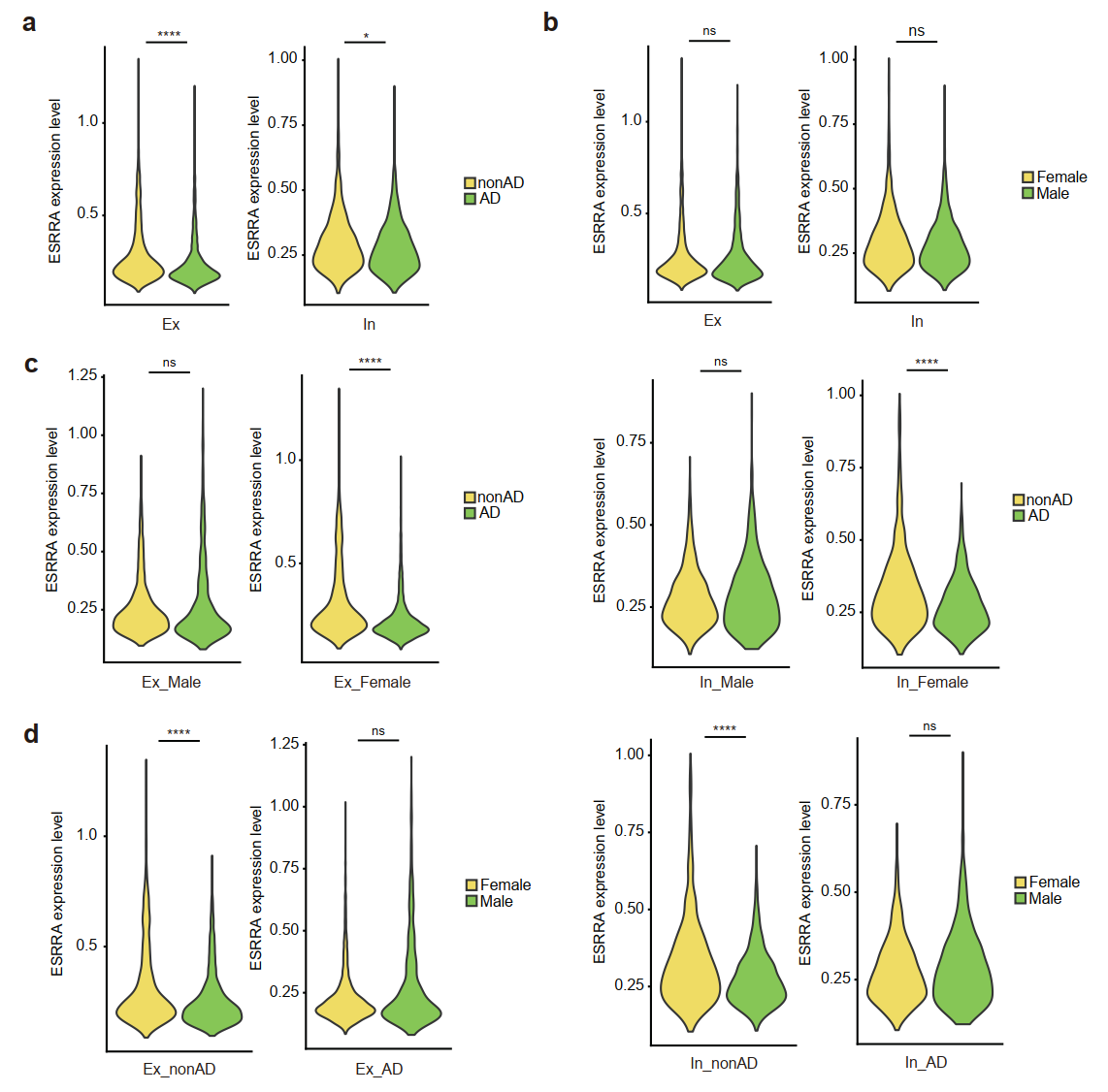
**

**Fig. S5:** **ESRRA expression stratified by AD pathology and gender**

1. Violin plots revealed differences in recovered ESRRA expression between individuals with non-AD pathology and AD pathology in excitatory and inhibitory neurons.
2. Violin plots revealed differences in recovered ESRRA expression between male and female in excitatory and inhibitory neurons.
3. Violin plots revealed differences in recovered ESRRA expression between male and female in excitatory and inhibitory neurons stratified by AD pathology.
4. Violin plots revealed differences in recovered ESRRA expression between individuals with non-AD pathology and AD pathology in excitatory and inhibitory neurons stratified by gender.


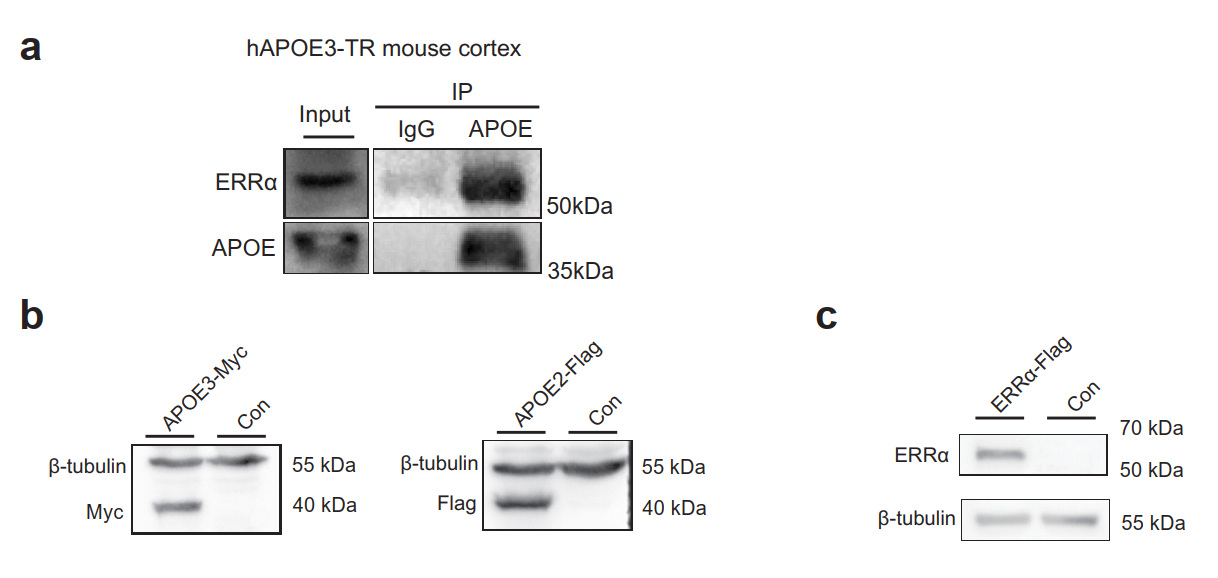


**Fig. S6: Detection of the binding between ERRα and ApoE** **in hAPOE3-TR mouse cortex and plasmid validation**

a. Co-IP indicating the direct bind of ERRα protein and ApoE3 protein in hAPOE3-TR mouse cortex.

b. Plasmid validation of APOE3-Myc and APOE2-Flag.

c. Plasmid validation of ESRRA-Flag.


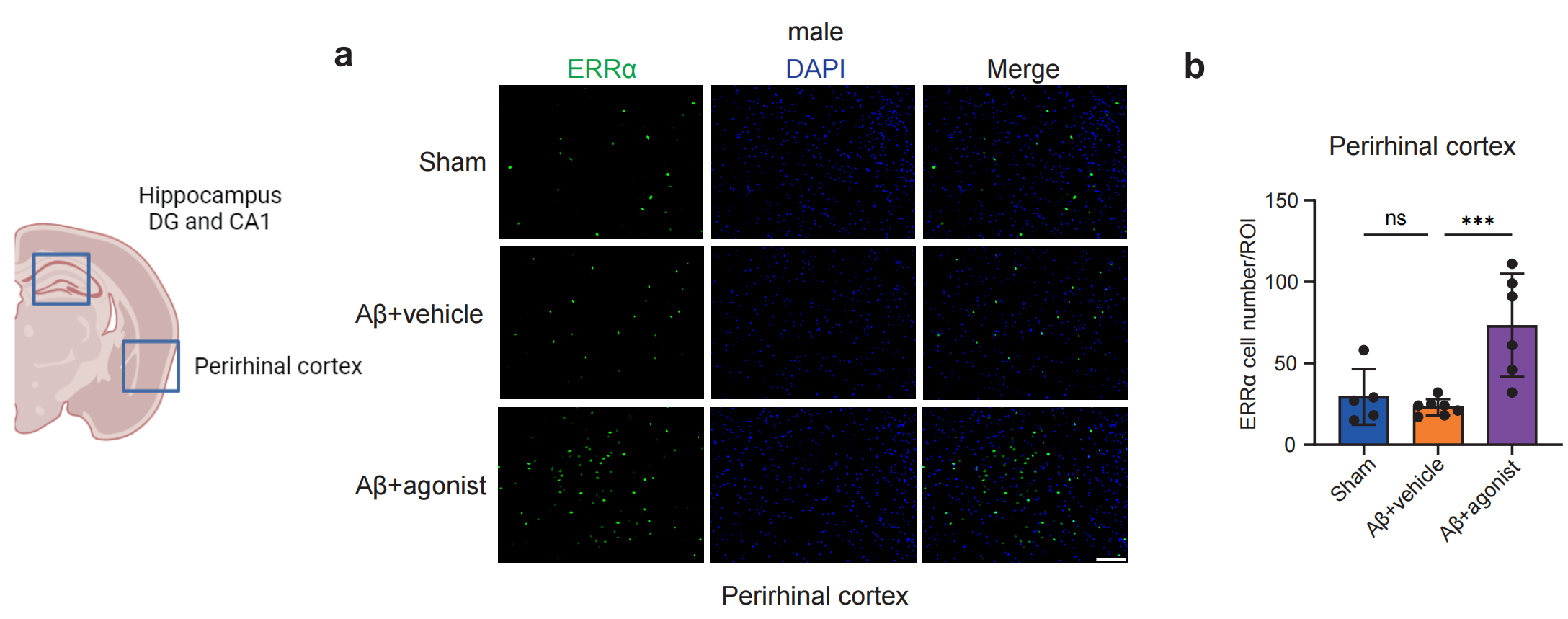


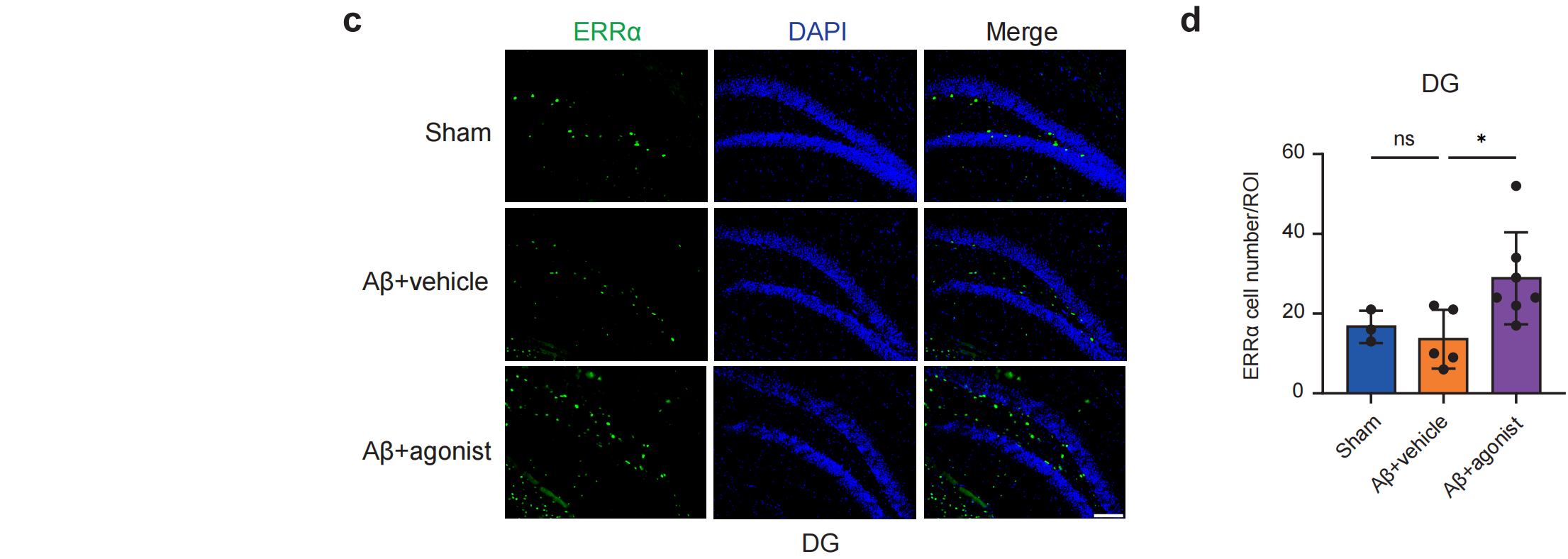


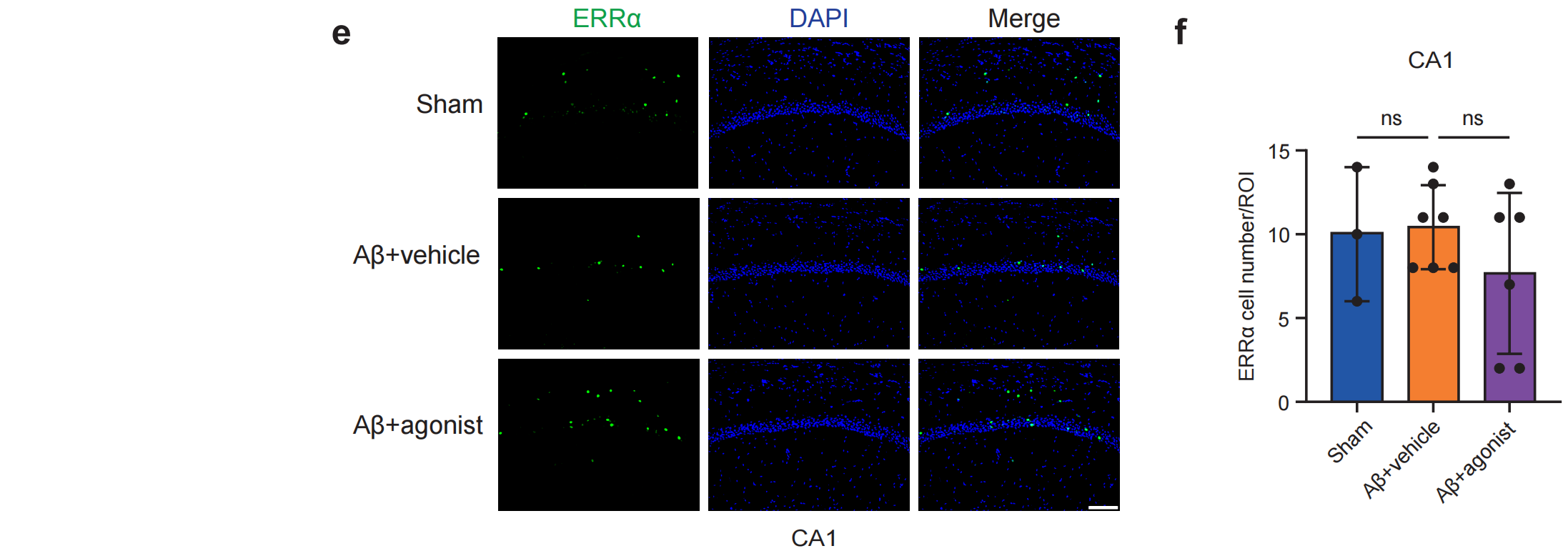


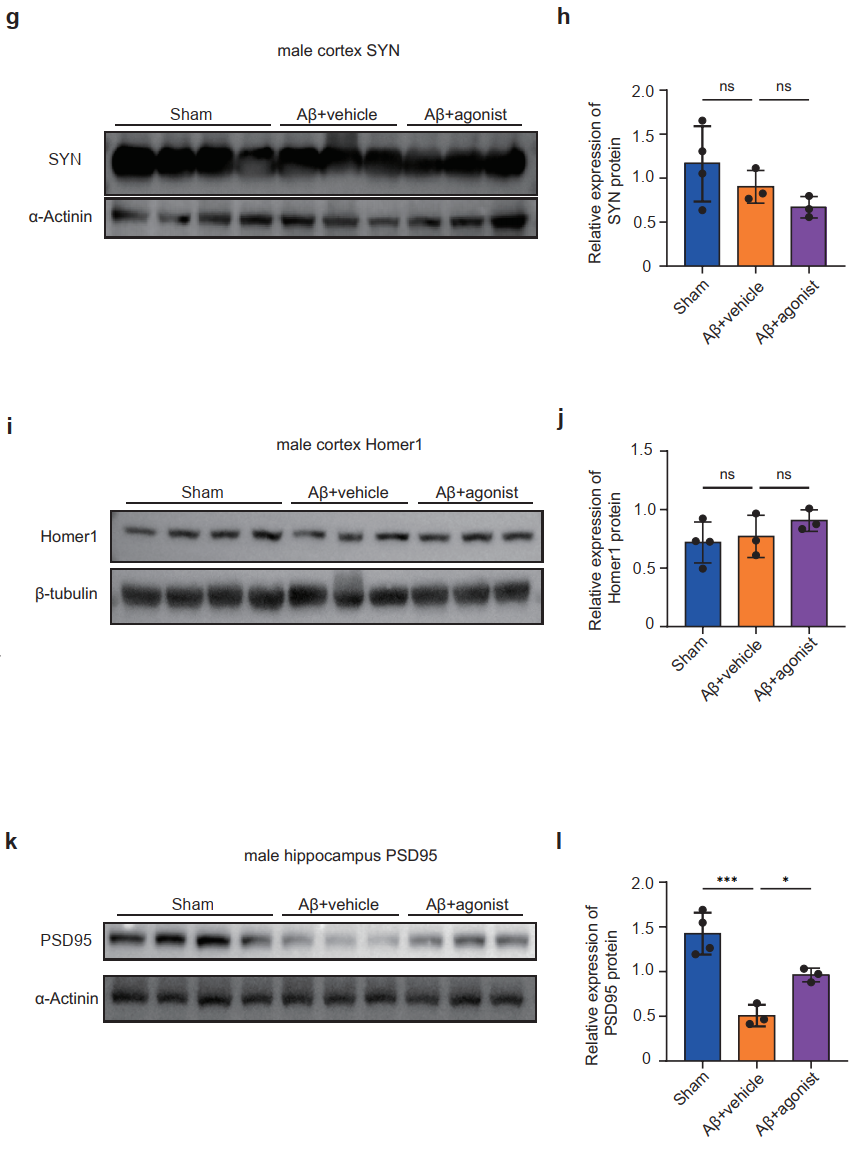


**Fig. S7: ERRα expression and synaptic molecular alterations following treatment with an** **ERRα agonist.**

1. Representative immunofluorescence staining of ERRα in perirhinal cortex. Scale bar, 100 μm.
2. Quantification analysis of ERRα positive cell numbers in perirhinal cortex (3–4 mice for each group).
3. Representative immunofluorescence staining of ERRα in DG regions. Scale bar, 100 μm.
4. Quantification analysis of ERRα positive cell numbers in DG (3-4 male mice for each group).
5. Representative immunofluorescence staining of ERRα in CA1 regions. Scale bar, 100 μm.
6. Quantification analysis of ERRα positive cell numbers in CA1 (3-4 mice for each group).
7. Representative immunoblotting images of SYN protein expression from male cortex.
8. Quantification analysis of ccortex SYN protein expression in male mice (n = 4, sham group; n = 3, Aβ+vehicle group; n = 3, Aβ+agonist group).
9. Representative immunoblotting images of Homer1 protein expression from male cortex.
10. Quantification analysis of cortex Homer1 protein expression in male mice (n = 4, sham group; n = 3, Aβ+vehicle group; n = 3, Aβ+agonist group).
11. Representative immunoblotting images of PSD95 protein expression from male hippocampus.
12. Quantification analysis of hippocampus PSD95 protein expression in male mice (n = 4, sham group; n = 3, Aβ+vehicle group; n = 3, Aβ+agonist group).

(b, d, f, h, j, l) Data were presented as the mean ± S.E; One-way ANOVA; *, P<0.05; ***, P<0.001; ns, no significant.
